# Supplementary material for: Controlling Epithelial Polarity: A Human Enteroid Model for Host-Pathogen Interactions
Source: Cell Rep. 2019 Feb 26;26(9):2509–2520.e4. doi: 10.1016/j.celrep.2019.01.108 (PMC6391775; doi:10.1016/j.celrep.2019.01.108)
Supplement: Document S1. Figures S1–S7 [file mmc1.pdf]

Cell Reports, Volume *26*

Supplemental Information

Controlling Epithelial Polarity:

A Human Enteroid

Model for Host-Pathogen Interactions

Julia Y. Co, Mar Margalef-Català, Xingnan Li, Amanda T. Mah, Calvin J. Kuo, Denise M. Monack, and Manuel R. Amieva

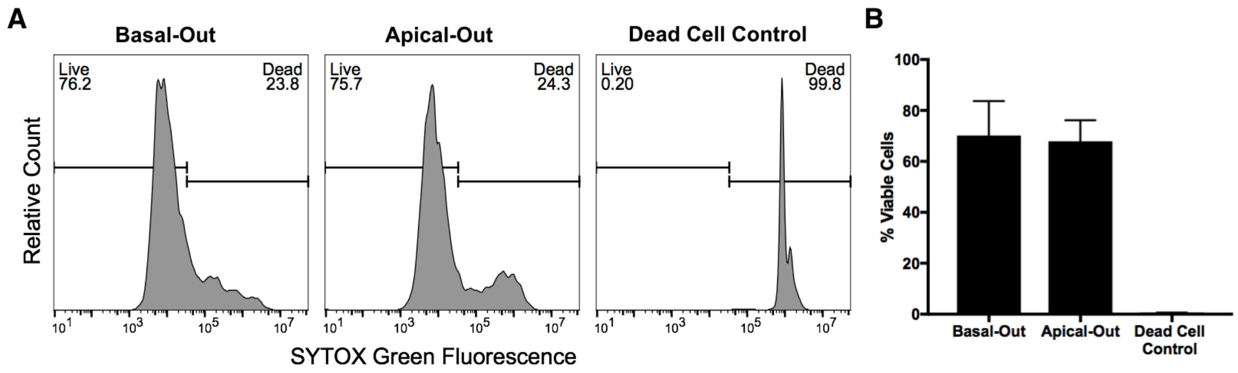

Figure S1. Flow cytometry analysis shows comparable cell viability for basal-out and apical-out enteroids, related to Figure 1. (**A**) Representative histograms of basal-out enteroid cells, apical-out enteroid cells, or ethanol-killed enteroid cells labeled with SYTOX Green dead cell stain. (**B**) Quantification of basal-out enteroid cells, apical-out enteroid cells, or ethanol-killed enteroid cells labeled with SYTOX Green dead cell stain. Data represented are mean ± SD; n = 3 experiments.

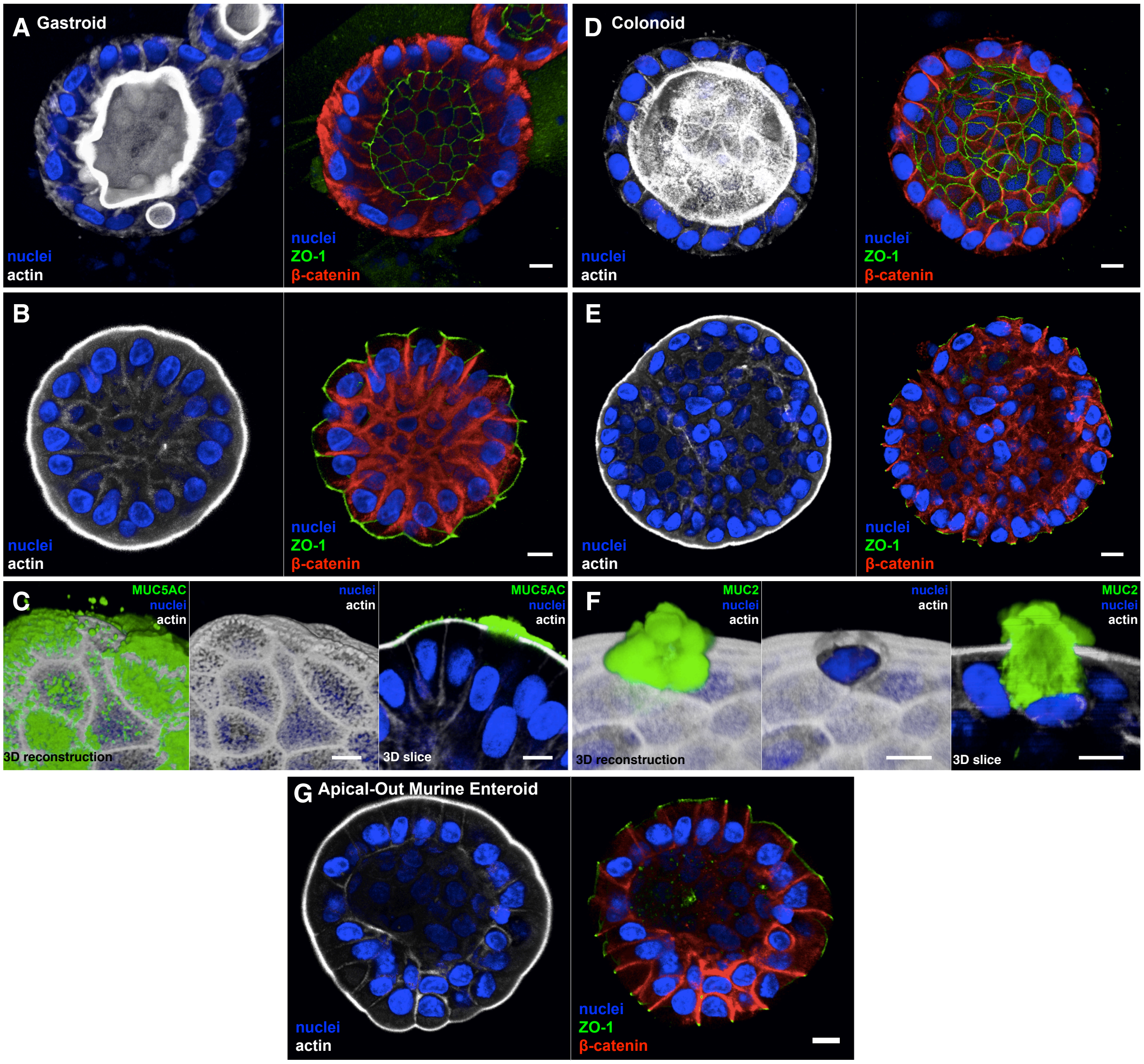

Figure S2. Polarity reversal occurs in human gastroids, human colonoids, and murine enteroids, related to Figure 1. Confocal microscopy shows that (**A**) BME-embedded gastroids exhibit basal-out polarity and (**B**) suspended gastroids have apical-out polarity. Nuclei in blue, actin in white, ZO-1 in green, and β-catenin in red are shown. (**C**) Apical-out gastroid cells produce the gastric mucin MUC5AC (green). (**D**) BME-embedded colonoids have basal-out polarity and (**E**) suspended colonoids have apical-out polarity. Nuclei in blue, actin in white, ZO-1 in green, and β-catenin in red are shown. (**F**) Apical-out colonoids can differentiate to goblet cells which secrete the intestinal mucin MUC2 (green). (**G**) Murine enteroids in suspension culture have apical-out polarity. Nuclei in blue, actin in white, ZO-1 in green, and β-catenin in red are shown. All scale bars are 10 μm.

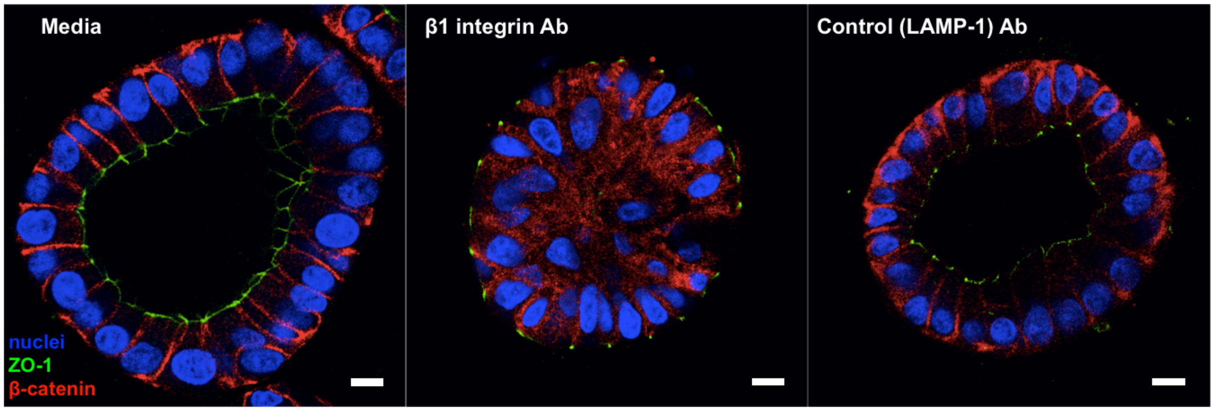

Figure S3. β1-integrin regulates enteroid polarity, related to Figure 2. Representative confocal images of BME-embedded enteroids incubated in media alone, with β1-integrin function-blocking antibody, or a control antibody for 1 day. Nuclei in blue, actin in white, ZO-1 in green, and β-catenin in red are shown. Scale bars are 10 µm.

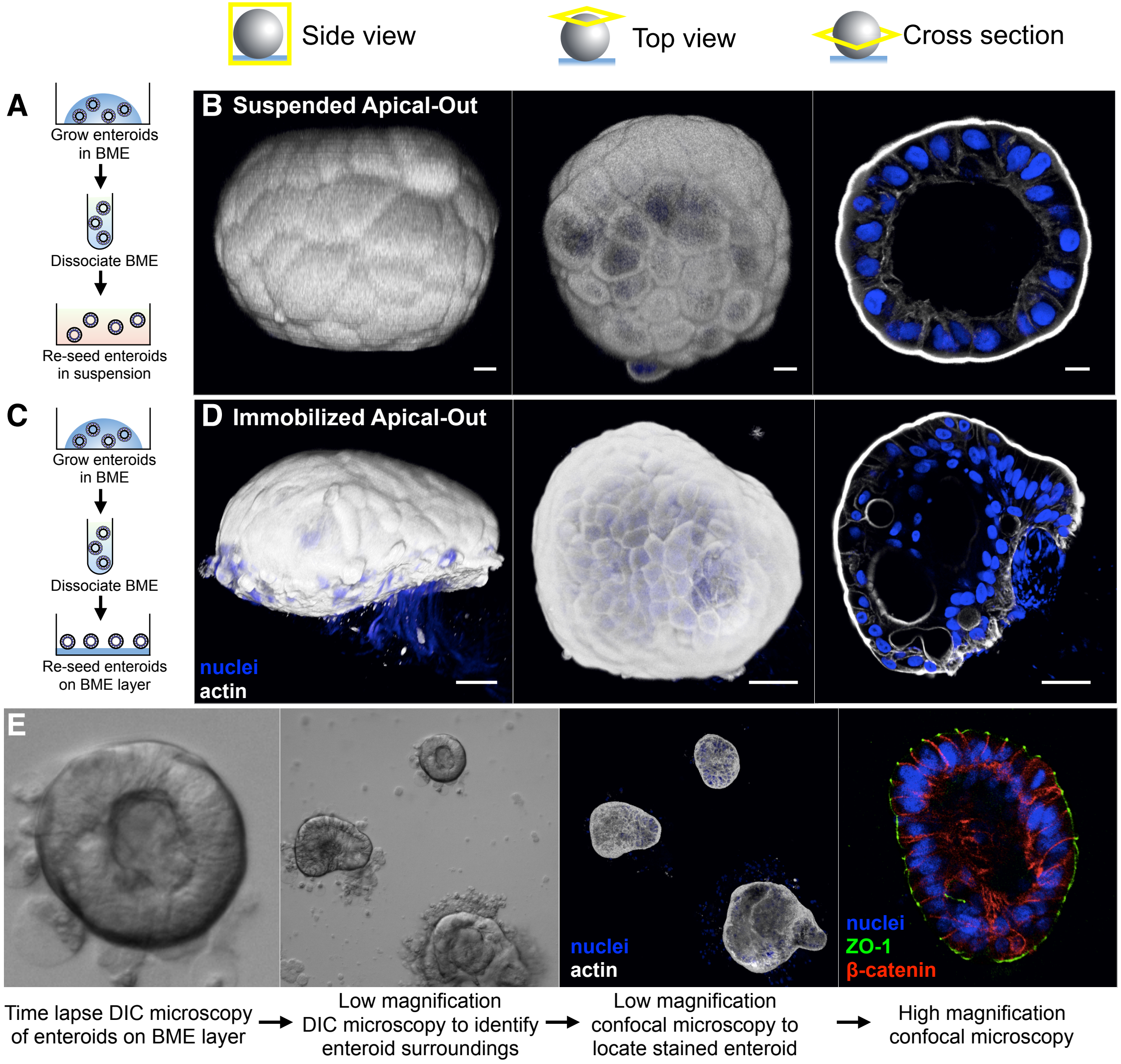

Figure S4. Immobilized apical-out enteroids for retrospective fluorescence time-lapse microscopy, related to Figure 1, 2. Comparison of (**A, B**) suspended and (**C**, **D**) immobilized apical-out enteroids. (**A**) Schematic for generating suspended apical-out enteroids. (**B**) Confocal microscopy of suspended enteroids, which have complete apical-out polarity. Scale bars are 10 µm. (**C**) Schematic to produce immobilized apical-out enteroids. (**D**) Confocal microscopy of immobilized apical-out enteroids, which have apical-out polarity except at the regions where the enteroid is in contact with the BME. Scale bars are 50 µm. (**E**) Workflow for retrospective fluorescence time-lapse microscopy. An enteroid is first imaged using time-lapse DIC microscopy, located within its surroundings, then fixed, stained and located using confocal microscopy, and finally imaged by confocal microscopy at higher magnification. For confocal images, nuclei in blue, actin in white, ZO-1 in green, and β-catenin in red are shown.

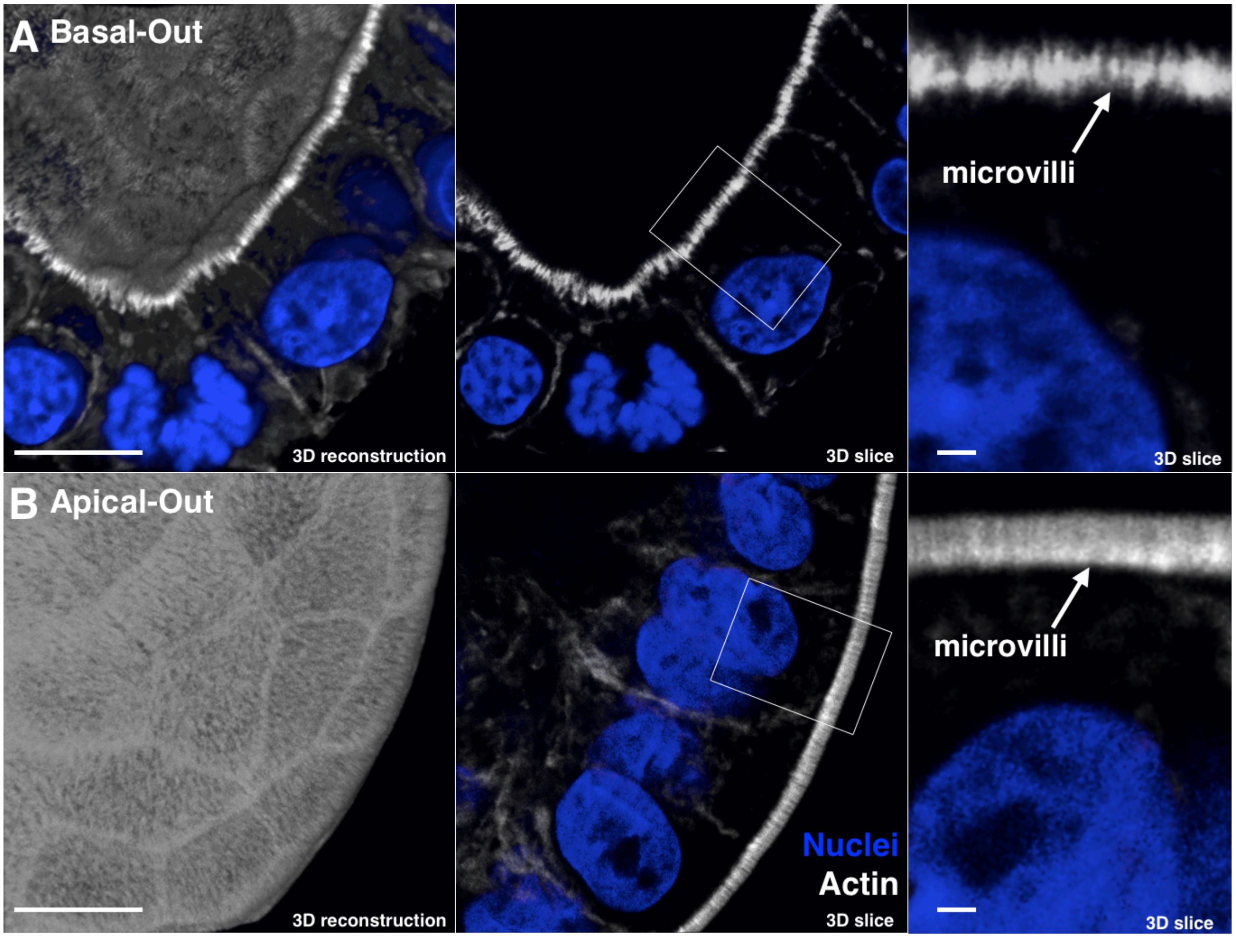

Figure S5. Apical microvillar structures of basal-out enteroids and apical-out enteroids, related to Figure 4. **(A)** Confocal microscopy of a BME-embedded basal-out enteroid shows the actin-rich apical microvilli are on the lumenal surface of the enteroid. **(B)** For apical-out enteroids, the actin-rich microvilli are on the outer enteroid surface. Left panels are 3D reconstructions and center panels are 3D slices through the cells (scale bars are 10 µm). Right panels are magnified insets of the middle panels (scale bars are 1 µm). Nuclei in blue and actin in white are shown.

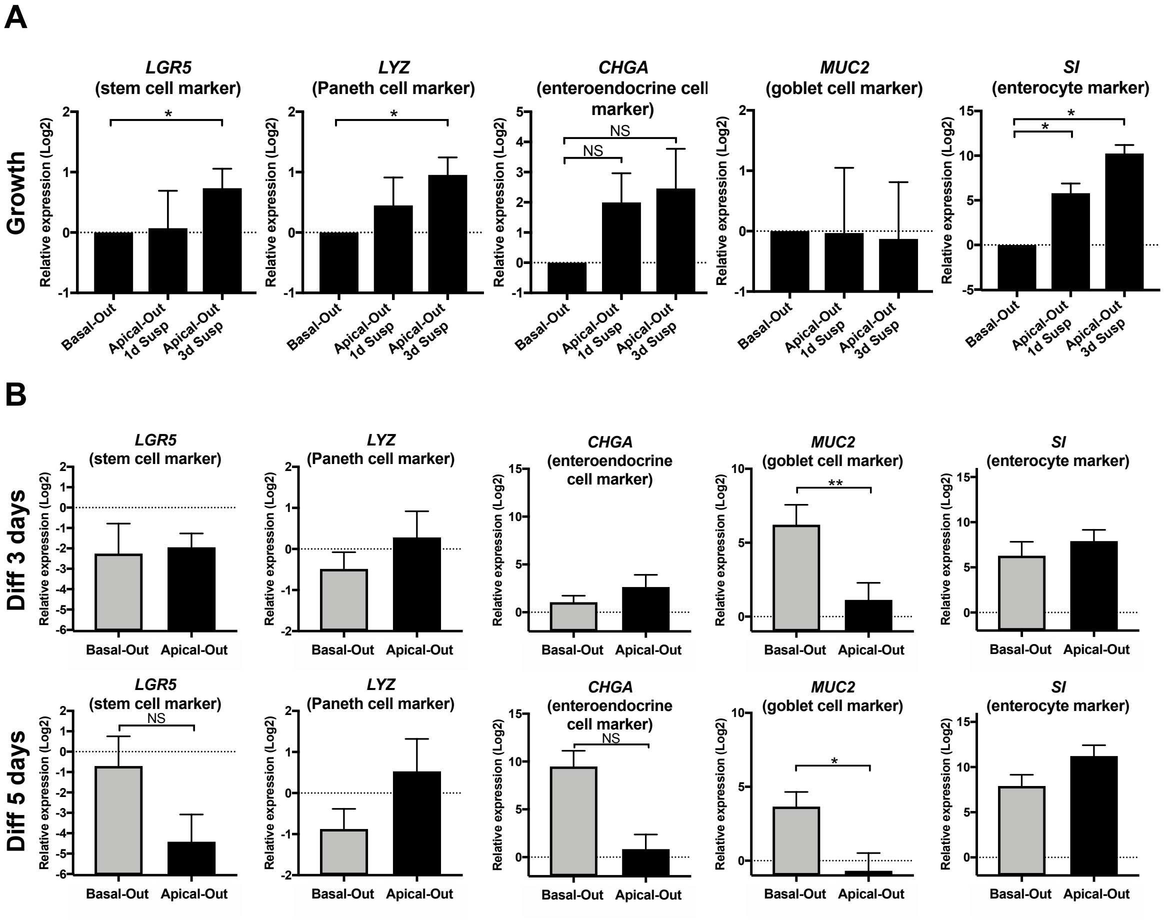

Figure S6. qRT-PCR gene expression analysis of enteroids, related to Figure 4. Expression of stem cell marker *LGR5* (which encodes leucine-rich repeat-containing G-protein coupled receptor 5), Paneth cell marker *LYZ* (which encodes lysozyme), enteroendocrine marker *CHGA* (which encodes chromagranin A), goblet cell marker *MUC2* (which encodes mucin 2), and enterocyte marker *SI* (which encodes sucrase isomaltase) was evaluated for enteroids cultivated in the following conditions: (**A**) In growth media, BME-embedded basal-out enteroids or suspended apical-out enteroids cultured for 1 or 3 days. Values are reported as fold-change relative to expression in basal-out enteroids cultured in growth media (dotted lines). Data represented are mean ± SD; $n \geq 3$ experiments; *$p < 0.05$ using the Kruskal-Wallis test with Dunn's multiple comparison test. (**B**) In differentiation media, BME-embedded basal-out enteroids or suspended apical-out enteroids cultured for 3 days or 5 days. Values are reported as fold-change relative to expression in basal-out enteroids cultured in growth media (dotted lines). Data represented are mean ± SD; $n \geq 4$ experiments; *$p < 0.05$, **$p < 0.05$ using the Mann-Whitney U test.

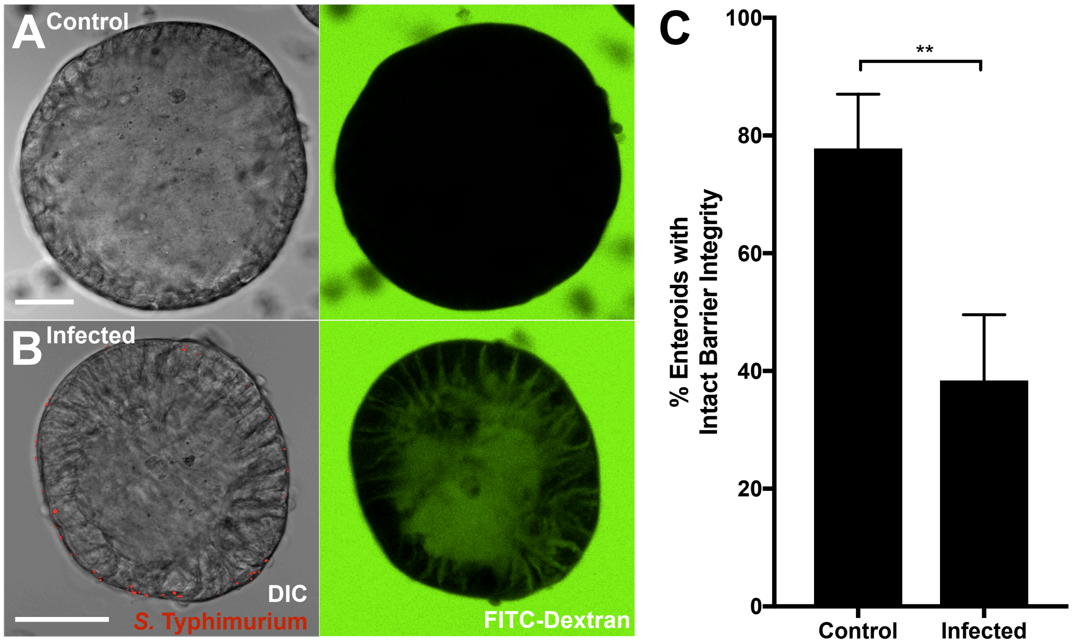

Figure S7. *S.* Typhimurium infection disrupts epithelial barrier integrity in apical-out differentiated enteroids, related to Figure 6. The dextran diffusion assay was used to evaluate uninfected (control) apical-out enteroids, or apical-out enteroids infected with *S.* Typhimurium for 1 hour. (**A**) An uninfected (control) apical-out enteroid that has intact barrier integrity. (**B**) An infected apical-out enteroid with disrupted barrier integrity. Scale bars are 50 µm. (**C**) Quantification of uninfected (control) or infected enteroids that exclude FITC-dextrans and thus have intact barrier integrity. Data represented are mean ± SD; n = 5 experiments; $p < 0.01$ using the Mann-Whitney U test.
