# Supplementary material for: Controlling Epithelial Polarity: A Human Enteroid Model for Host-Pathogen Interactions
Source: Cell Rep. 2019 Feb 26;26(9):2509–2520.e4. doi: 10.1016/j.celrep.2019.01.108 (PMC6391775; doi:10.1016/j.celrep.2019.01.108)
Supplement: Document S2. Article plus Supplemental Information [file mmc4.pdf]

Cell Reports

Resource

Controlling Epithelial Polarity: A Human Enteroid Model for Host-Pathogen Interactions

Graphical Abstract

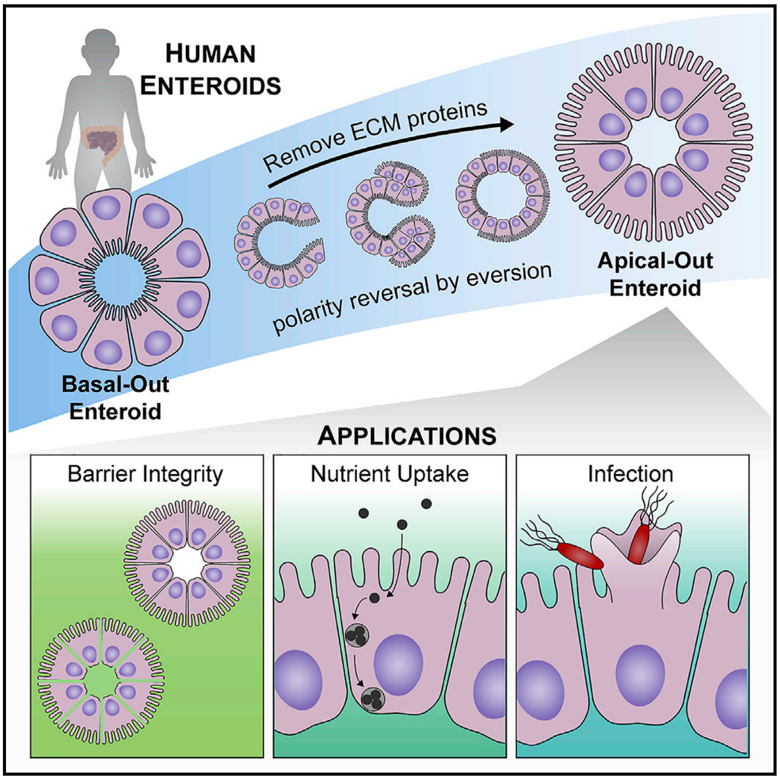

Authors

Julia Y. Co, Mar Margalef-Català, Xingnan Li, Amanda T. Mah, Calvin J. Kuo, Denise M. Monack, Manuel R. Amieva

Correspondence

amieva@stanford.edu

In Brief

Co et al. describe a method to reverse human enteroid polarity, thus enabling access to the apical epithelium. Upon removing ECM scaffold proteins, basal-out enteroids evert to apical-out polarity in a β1 integrin-dependent manner. Demonstrated applications for this model include evaluating barrier integrity, monitoring nutrient uptake, and examining bacterial infection.

Highlights

- Enteroid polarity reversal by ECM protein removal enables apical epithelial access
- Basal-out enteroids evert to apical-out polarity in a β1 integrin-dependent manner
- Apical-out enteroids differentiate to the major intestinal epithelial cell types
- An effective model to probe barrier integrity, nutrient uptake, and infection

Co et al., 2019, Cell Reports *26*, 2509–2520
February 26, 2019 © 2019 The Author(s).
https://doi.org/10.1016/j.celrep.2019.01.108

OPEN ACCESS
CellPress

Controlling Epithelial Polarity: A Human Enteroid Model for Host-Pathogen Interactions

Julia Y. Co,[1] Mar Margalef-Català,[1] Xingnan Li,[2] Amanda T. Mah,[2] Calvin J. Kuo,[2] Denise M. Monack,[3] and Manuel R. Amieva[1,3,4,*]
[1]Department of Pediatrics, Division of Infectious Diseases, Stanford University, Stanford, CA 94305, USA
[2]Department of Medicine, Division of Hematology, Stanford University, Stanford, CA 94305, USA
[3]Department of Microbiology and Immunology, Stanford University, Stanford, CA 94305, USA
[4]Lead Contact
*Correspondence: amieva@stanford.edu
https://doi.org/10.1016/j.celrep.2019.01.108

SUMMARY

Human enteroids—epithelial spheroids derived from primary gastrointestinal tissue—are a promising model to study pathogen-epithelial interactions. However, accessing the apical enteroid surface is challenging because it is enclosed within the spheroid. We developed a technique to reverse enteroid polarity such that the apical surface everts to face the media. Apical-out enteroids maintain proper polarity and barrier function, differentiate into the major intestinal epithelial cell (IEC) types, and exhibit polarized absorption of nutrients. We used this model to study host-pathogen interactions and identified distinct polarity-specific patterns of infection by invasive enteropathogens. *Salmonella enterica* serovar Typhimurium targets IEC apical surfaces for invasion via cytoskeletal rearrangements, and *Listeria monocytogenes*, which binds to basolateral receptors, invade apical surfaces at sites of cell extrusion. Despite different modes of entry, both pathogens exit the epithelium within apically extruding enteroid cells. This model will enable further examination of IECs in health and disease.

INTRODUCTION

The intestinal epithelium is the barrier that mediates interactions between the intestinal lumen and the rest of the body. Proper intestinal function depends on the development and maintenance of organization of epithelial cells into two distinct compartments, the apical and basolateral epithelial regions. The apical surface of intestinal epithelial cells (IECs) faces the lumen and regulates interactions with lumenal contents. For example, the apical surface mediates nutrient absorption, detects microbial products, and secretes molecules that protect the epithelium from potentially harmful agents in the lumen. The basolateral surface anchors epithelial cells to the underlying basement membrane, delivers nutrients from the lumen to the bloodstream, and communicates with nearby cells. The establishment of polarity requires the formation of apical junctional complexes and proper compartmentalization of apical and basolateral proteins. In addition to proper polarity, intestinal epithelial function relies on differentiation into the various IEC types, which each carry out specific processes. Due to its complexity, it has been challenging to model the human intestinal epithelium. Transformed cell lines have enabled investigation of epithelial cells *in vitro*; however, these cancer-derived cultures do not differentiate into the various IEC types and often do not develop proper polarity. Although animal models have provided valuable insights to intestinal function and disorders, these systems often do not recapitulate human-specific infections and diseases.

Human organoids have recently emerged as a promising system to model the human gastrointestinal tract (Sato et al., 2009; Spence et al., 2011). Gastrointestinal organoids are cell spheroids that can differentiate into the various gastrointestinal cell types. These patient-derived cell culture systems not only provide a physiologically relevant model but also allow the investigation of individual variation in intestinal function, drug and therapeutic efficacy, and interactions with microbes. Human intestinal organoids cultivated from pluripotent stem cells (either embryonic stem cells or induced pluripotent stem cells) require several weeks to develop and form both epithelial and mesenchymal lineages (Spence et al., 2011). Alternatively, organoids cultivated from biopsies or surgical specimens of intestinal tissue can be derived from crypt stem cells within a few days and are comprised of entirely epithelial lineages (Miyoshi and Stappenbeck, 2013; Sato et al., 2009). These epithelial-only organoids are also called enteroids.

The development of the human enteroid model has led to novel discoveries about human intestinal biology. These advances include our understanding of human-specific infections. Researchers have used human enteroid models to investigate the pathogenesis of rotavirus (Saxena et al., 2015), adenovirus (Holly and Smith, 2018), *Salmonella* (Zhang et al., 2014), and pathogenic *Escherichia coli* strains (Rajan et al., 2018; VanDussen et al., 2015). Human enteroids have even enabled *in vitro* study of norovirus (Ettayebi et al., 2016), which was previously impossible using cell lines in culture.

This is an open access article under the CC BY license (http://creativecommons.org/licenses/by/4.0/).

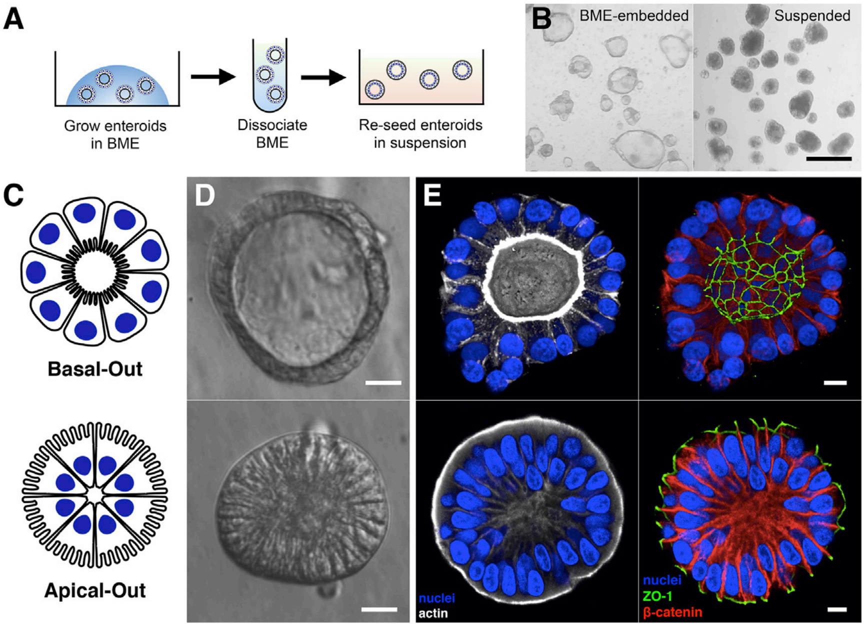

Figure 1. Enteroids in Suspension Culture Exhibit Apical-Out Polarity

(A) Schematic for generation of suspended apical-out enteroids.

(B) Images from a dissection microscope of BME-embedded enteroids (left) or suspended enteroids (right). Scale bar is 500 μm.

(C–E) Basal-out enteroids and apical-out enteroids are (C) depicted schematically, (D) imaged using modulation contrast microscopy, and (E) imaged using confocal microscopy. Nuclei in blue, actin in white, ZO-1 in green, and β-catenin in red are shown. Scale bars are 10 μm.

See also Figures S1, S2, and S4.

A challenge in using organoids or enteroids to study epithelial interactions with lumenal contents, such as nutrients or microbes, is that the apical surface of the epithelium is enclosed within the spheroid and therefore difficult to access. Most studies have employed microinjection techniques to introduce microbes and agents of interest into the spheroid lumens (Bartfeld and Clevers, 2015; Bartfeld et al., 2015); however, this is a slow and labor-intensive task and results can be confounded by the accessibility of the epithelial surface due to accumulation of mucus and cell debris within the enclosed enteroid or organoid lumen. Others have studied intestinal monolayers by seeding dissociated enteroid cells onto Transwell permeable supports (VanDussen et al., 2015). This 2D culture method allows independent control of apical and basolateral surfaces but requires a large number of cells and is difficult to visualize by microscopy without Transwell system disassembly.

We have developed an enteroid cultivation technique that maintains the 3D spheroid structure while making the apical surface accessible to experimental challenges. We devised a method to reverse the epithelial polarity of the enteroids such that the apical surface faces outward. By manipulating extracellular matrix (ECM) proteins in the culture system, we successfully produced apical-out enteroids, which maintain their ability to differentiate to the various IEC lineages, maintain proper polarity and barrier function, and are able to absorb nutrients in a polarity-specific manner. This method bypasses the need for microinjection because compounds or microbes can be simply added to the culture media to interact with the apical enteroid surface. Here, we show that this model can be used to recapitulate and advance our understanding of intestinal pathogens and host-microbe interactions. We propose that apical-out human enteroids can be utilized for a broad range of applications beyond those demonstrated here.

RESULTS

Development of the Apical-Out Enteroid Model

The original human enteroid cultivation system based on maintaining Lgr5+ intestinal stem cells uses Matrigel or its equivalent basement membrane extract (BME), which are comprised of ECM proteins, as a scaffold that encases the 3D spheroids (Sato et al., 2009). The enteroids form with their basolateral epithelial surfaces, which are in contact with the BME, facing outward (Figures 1C–1E). The enteroid apical epithelial surfaces, and thus the "lumen," are in the spheroid interior. This model presents a challenge to study experimental interactions between the apical surface of the epithelium with lumenal contents, such as nutrients and microbes, because the spheroids are a closed system. We hypothesized that we could reverse the polarity of the enteroids such that the apical surface faces outward by manipulating ECM components in the culture system. Studies using Madin-Darby canine kidney (MDCK) polarized epithelial spheroids previously demonstrated that ECM proteins regulate epithelial polarity (Wang et al., 1990), specifically that MDCK spheroids in a collagen gel exhibit basal-out polarity, and that spheroids in suspension without ECM proteins exhibit apical-out polarity. We hypothesized that, if enteroids are first grown embedded within BME and then removed from BME without dissociating them into single cells, the enteroid polarity could be reversed. To test this, we derived enteroids from healthy human intestinal tissue and grew them embedded within BME. To maintain enteroid integrity during removal from BME, the chelator EDTA was used to disrupt divalent cation-dependent polymerization of the ECM protein laminin (Yurchenco et al., 1985), a primary component of BME. Enteroids were transferred to suspension culture in growth media using low-attachment plates (Figure 1A). After 3 days in suspension culture, examination on a dissection microscope (Figure 1B) or by modulation contrast microscopy (Figure 1D) showed that the enteroid morphology changes. Although BME-embedded enteroids maintain a clear central lumen, the suspension enteroids often lack a lumen and the edges of columnar epithelial cells become visible. Confocal 3D immunofluorescence imaging of apical protein ZO-1 and basolateral protein β-catenin revealed that suspended enteroids indeed have

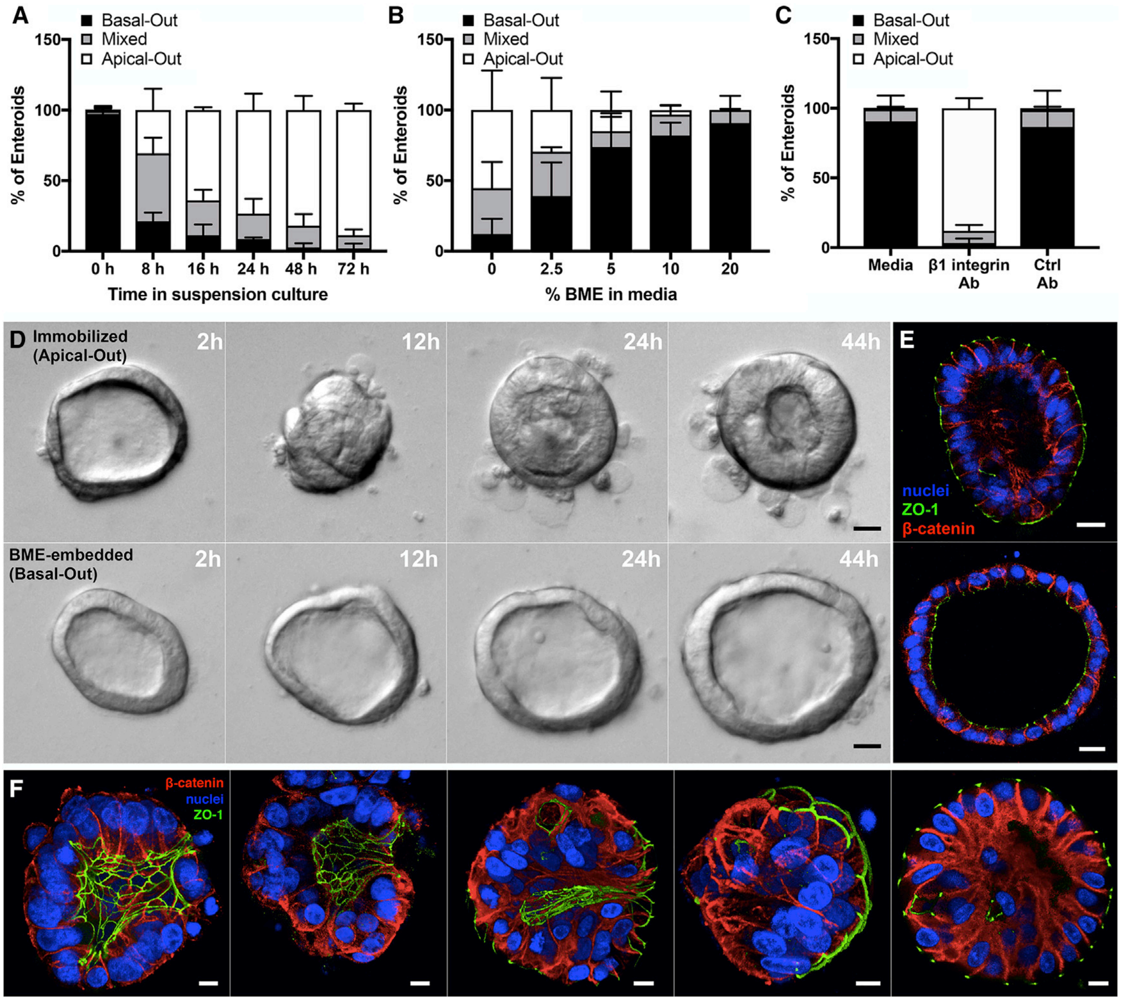

Figure 2. Characterization of Enteroid Polarity Reversal

(A) Enteroids were analyzed using confocal microscopy and quantified for percentage of basal-out, apical-out, or mixed polarity enteroids; n = 3 experiments.

(B) Quantification of basal-out, apical-out, or mixed polarity enteroids in suspension culture with soluble BME; n = 3 experiments.

(C) BME-embedded enteroids were incubated in media alone or with β1-integrin function-blocking antibody or a control antibody for 1 day; n = 3 experiments. For (A)–(C), data represented are the means of each category with SD.

(D) Time-lapse DIC microscopy of immobilized apical-out (top) or BME-embedded basal-out (bottom) enteroids as shown in Video S1.

(E) Confocal microscopy of enteroids from time-lapse experiment. Nuclei in blue, ZO-1 in green, and β-catenin in red are shown. Scale bars are 20 μm.

(F) Confocal microscopy of suspended enteroids at different stages of polarity reversal. Nuclei in blue, ZO-1 in green, and β-catenin in red are shown. Scale bars are 10 μm.

See also Figures S3 and S4 and Video S1.

reversed polarity such that the apical surfaces face outward (herein called apical-out enteroids), and BME-embedded enteroids have basolateral surfaces facing outward (herein called basal-out enteroids; Figure 1E). Strong actin staining of microvilli brush borders confirmed that the apical surfaces of suspended enteroids face outward (Figure 1E). Cell viabilities for basal-out and apical-out enteroids were comparable, as measured by flow cytometry analysis of cells from dissociated enteroids stained with SYTOX Green (Figure S1). The polarity reversal method can be applied to other segments of the gastrointestinal tract, because gastroids derived from primary human gastric tissue and colonoids derived from primary human colon tissue both have basal-out polarity when embedded within BME and acquire apical-out polarity after transfer to suspension cultures (Figure S2). Murine enteroids also exhibit apical-out polarity after transfer from BME to suspension culture (Figure S2).

Characterization of Enteroid Polarity Reversal

To understand the kinetics of enteroid polarity reversal, we used confocal microscopy to quantitate the percentage of enteroids with apical-out, basal-out, or mixed (partial apical-out and partial basal-out) polarity in a time course experiment (Figure 2A). This revealed that enteroid polarity reversal occurs within as early as 8 h after transfer to suspension culture. After the first day of

suspension culture, the majority of enteroids already have apical-out or mixed polarity. By the third day in suspension culture, almost all enteroids have apical-out polarity.

The removal of BME from enteroid cultures results in polarity reversal and the generation of apical-out polarity. These results, taken together with the known role of ECM proteins in regulating MDCK spheroid polarity, suggest that interactions with ECM proteins determine enteroid polarity. To test this, BME-embedded enteroids were isolated and re-suspended in growth media containing titrated concentrations of soluble BME. After 3 days, the enteroids were analyzed by confocal microscopy to quantify the frequency of apical-out enteroids. The percentage of apical-out enteroids decreases as the concentrations of BME in the cultures increase (Figure 2B). This dose-dependent phenotype supports the hypothesis that ECM proteins regulate enteroid polarity reversal.

ECM proteins are known ligands for the basolateral receptor β1 integrin, which in MDCK spheroids is important to control the orientation of epithelial polarity. Previous work showed that treatment with a β1 integrin function-blocking antibody can inhibit polarity reversal that occurs when MDCK spheroids in suspension are transferred into a collagen gel (Ojakian and Schwimmer, 1994). Additionally, treatment of collagen-embedded MDCK spheroids with this blocking antibody caused polarity reversal from basal-out to apical-out polarity (Yu et al., 2005). We hypothesized that β1 integrin signaling is also necessary to regulate human enteroid polarity. BME-embedded enteroids were treated with the β1 integrin function-blocking antibody and harvested after 1 day to examine enteroid polarity. In contrast to the BME-embedded enteroids treated with no antibody or a control antibody, which maintained basal-out polarity, the enteroids treated with the β1 integrin function-blocking antibody reversed to produce apical-out polarity (Figures 2C and S3). This finding indicates that β1 integrin signaling regulates the orientation of epithelial polarity in human enteroids.

Polarity modulation has been previously observed in several epithelial systems. In MDCK spheroids, polarity reversal occurs as a result of protein and organelle migration from one epithelial cell pole to the other (Wang et al., 1990). In segments of porcine thyroid follicles, epithelial sheets fold in an eversion-like process to produce "inside-out polar organization" (Graebert et al., 1997). We probed the process of human enteroid polarity reversal in more detail using time-lapse microscopy, which required that enteroids were immobilized. To produce immobilized apical-out enteroids, BME-embedded enteroids were isolated with EDTA treatment and then re-seeded on top of a thin BME layer to which the enteroids adhered (Figure S4). Similar to suspended enteroids, these immobilized enteroids develop apical-out polarity. However, these immobilized apical-out enteroids maintain basal-out polarity at the regions where the enteroid is in contact with the BME (Figure S4), consistent with the notion that ECM proteins determine polarity. Time-lapse differential interference contrast (DIC) microscopy showed that, after being seeded on top of the BME layer, the enteroids undergo a dramatic morphological rearrangement that results in eversion of the apical surface and polarity reversal (Figure 2D; Video S1). We observed that the enteroid everted as a unit, expelling its luminal contents and placing the apical side of the epithelium on the outside of the spheroid. Furthermore, the epithelial cells become more columnar. In contrast, BME-embedded enteroids exhibit some movement and shape changes; however, they maintain a lumen and do not undergo epithelial rearrangements (Figure 2D; Video S1). The enteroids were then analyzed using retrospective immunofluorescence confocal microscopy. In this method, the same enteroids observed using time-lapse microscopy were located by matching the surroundings, fixed and stained for actin, ZO-1, and β-catenin, and then imaged using confocal microscopy (Figure S4). This confirmed that the immobilized enteroids indeed have apical-out polarity and that BME-embedded enteroids maintain basal-out polarity (Figure 2E). Next, we examined enteroids that had been in suspension culture for 6–8 h. Enteroids at different stages of polarity reversal by eversion were observed by confocal microscopy (Figure 2F). This suggests that, similar to immobilized apical-out enteroids, the main mechanism of polarity reversal for suspended enteroids is also eversion.

Apical-Out Enteroids Maintain Epithelial Barrier Integrity

A key function of the intestinal epithelium is to provide a barrier separating the lumen from the underlying tissue. The apical tight junctions form an intercellular seal that prevents passage of material through the epithelial monolayer. To determine whether apical-out enteroids form a functional barrier, we performed a dextran diffusion assay. In this assay, enteroids were incubated in a solution of fluorescent dextrans (4 kDa fluorescein isothiocyanate [FITC]-dextrans) and then imaged using DIC and fluorescence confocal microscopy. We observed that the apical-out enteroids completely excluded the FITC-dextrans, as expected for epithelium with intact barrier integrity (Figure 3A). For comparison, apical-out enteroids were treated with EDTA, which disrupts tight junctions and results in compromised barrier integrity. Indeed, for the EDTA-treated apical-out enteroids, the FITC-dextrans diffused into the intercellular spaces and into the center of the spheroid (Figure 3B). These findings confirm that apical-out enteroids form an intact epithelial barrier and establish the dextran diffusion assay as a method to study epithelial barrier integrity in apical-out enteroids.

Apical-Out Enteroid Differentiation

One primary advantage of the human enteroid model is that the enteroid cells can differentiate into various IEC lineages. For BME-embedded basal-out enteroids, altering the growth factors in the media leads to differentiation from intestinal stem cells into the various types of IECs (Sato et al., 2011). BME-embedded enteroids were isolated and re-suspended in either growth media or differentiation media for comparison. Similar to suspension cultures in growth media, enteroids suspended in differentiation media reverse their polarity to an apical-out phenotype. Proliferating cells in enteroids were identified using immunofluorescence confocal microscopy for the proliferation marker Ki67 (Figure 4A) and were quantified as the percentage of Ki67-positive cells per enteroid (Figure 4B). As expected, both basal-out enteroids and apical-out enteroids had low percentages of proliferating cells when shifted to differentiation media for 3 days (1% and 9%, respectively) or 5 days (4% for apical-out

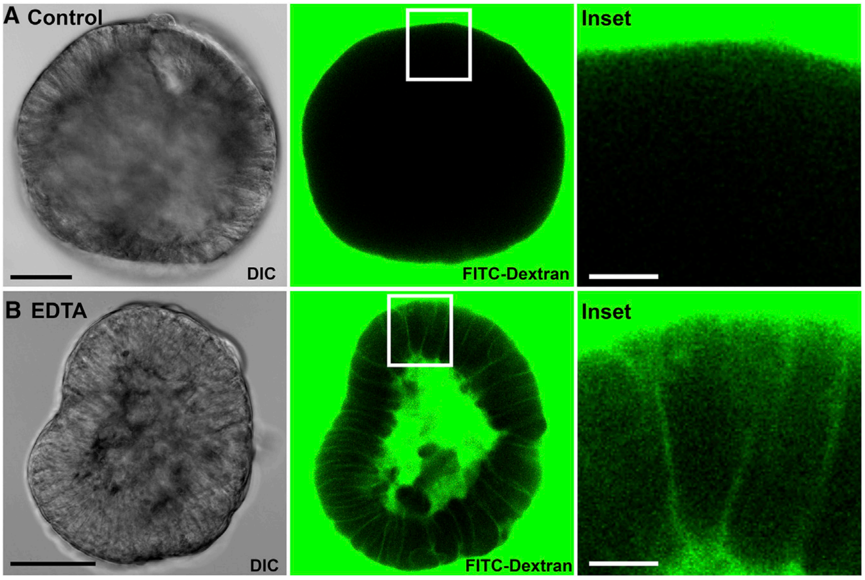

Figure 3. Dextran Diffusion Assay for Enteroid Epithelial Barrier Integrity

(A) Apical-out enteroids exclude FITC-dextrans added to the media, demonstrating intact epithelial barrier integrity.

(B) Treatment with 2 mM EDTA disrupts the epithelial barrier and results in diffusion of FITC-dextrans into the intercellular spaces and into the center of the spheroid.

Enteroids imaged by DIC microscopy (left; scale bars are 50 μm), FITC-dextran fluorescence imaged by confocal microscopy (middle), and insets of center (right; scale bars are 10 μm) are shown. See also Figure S7.

enteroids). When cultured in growth media, basal-out enteroids had an average of 77% proliferating cells. The percentage of proliferating cells in apical-out enteroids in growth media decreases over time, with 48% proliferating cells after 1 day and 26% after 3 days in suspension culture.

To visualize enteroid cell differentiation into the various IEC lineages, we performed confocal immunofluorescence microscopy. We show that cells in apical-out enteroids in differentiation media express markers for Paneth cells (lysozyme), goblet cells (MUC2), entero-endocrine cells (chromogranin A [CHGA]), or enterocytes (villin; Figure 4C). Additionally, actin-rich apical brush border microvilli could be observed on the outer surfaces of apical-out enteroids using confocal microscopy (Figure S5). Enteroids were further characterized using qRT-PCR to evaluate transcriptional expression of markers of the various IEC lineages, specifically stem cell marker *LGR5* (which encodes leucine-rich repeat-containing G-protein-coupled receptor 5), Paneth cell marker *LYZ* (which encodes lysozyme), enteroendocrine marker *CHGA* (which encodes chromogranin A), goblet cell marker *MUC2* (which encodes mucin 2), and enterocyte marker *SI* (which encodes sucrase isomaltase). After 1 day of suspension culture in growth media, apical-out enteroids showed similar expression levels for *LGR5*, *CHGA*, *LYZ*, and *MUC2* and higher levels of *SI* compared to basal-out enteroids (Figure S6). After 3 days in suspension culture in growth media, apical-out enteroids continue to increase *SI* expression and have slightly elevated *LYZ* levels, suggesting that there is some differentiation occurring for apical-out enteroids in growth media. After being transferred to differentiation media, both basal-out enteroids and apical-out enteroids downregulated *LGR5* expression relative to basal-out enteroids in growth media (represented by dotted lines in graphs; Figure S6). The proportions of differentiated cell marker expression between basal-out and apical-out enteroids were equivalent except that apical-out enteroids had lower expression of MUC2, suggesting that there are fewer goblet cells.

Fatty Acid Uptake in Apical-Out Enteroids

As a functional test for polarity-specific processes, apical-out enteroids were evaluated for fatty acid uptake. Studies using animal models and cell lines have determined that fatty acids are absorbed through the apical surfaces of epithelial cells via interactions with fatty acid transporters, including CD36, plasma membrane-associated fatty acid-binding protein (FABP (pm)), and fatty acid transport proteins (FATP1–6; Wang et al., 2013). Once absorbed, fatty acids are incorporated into lipid droplets and then trafficked to the basolateral regions of the cells, where they are subsequently secreted (Wang et al., 2013). To evaluate fatty acid uptake, we used a fluorescent fatty acid analog, an established tool used to study lipid transport (Pagano and Sleight, 1985). Enteroids were incubated with the fatty acid analog C1-BODIPY-C12 for 30 min and then fixed, stained, and analyzed using confocal microscopy. Strong fluorescent signal was observed in apical-out enteroids, indicating that these cells can absorb the fatty acid analog from the media (Figure 5). We observed intracellular foci of fluorescent lipid droplets in the basal regions of these enteroid cells. In contrast, the fluorescent signal in basal-out enteroids was weaker and lipid droplets did not form, suggesting that the basal-out enteroids cannot readily absorb fatty acids from the media (Figure 5). This is consistent with the absence of accessible apical fatty acid transport proteins on the outer surface of basal-out enteroids.

Infection of Apical-Out Enteroids with *S.* Typhimurium

In addition to its role in polarized nutrient absorption, an important function of the intestinal epithelium is to mediate interactions with commensal microbes and potential pathogens that are transiting or residing in the lumen. *Salmonella enterica* serovar Typhimurium (*S.* Typhimurium) is a bacterial pathogen that can cause bacteremia in infants and immunocompromised humans (Coburn et al., 2007) and self-limiting inflammatory gastroenteritis with diarrhea in immunocompetent humans. This may be correlated with the ability of *S.* Typhimurium to disrupt epithelial barrier function in epithelial monolayers (Finlay and Falkow, 1990; Hecht, 1995). Consistent with these studies, we used the dextran

OPEN
ACCESS
CellPress

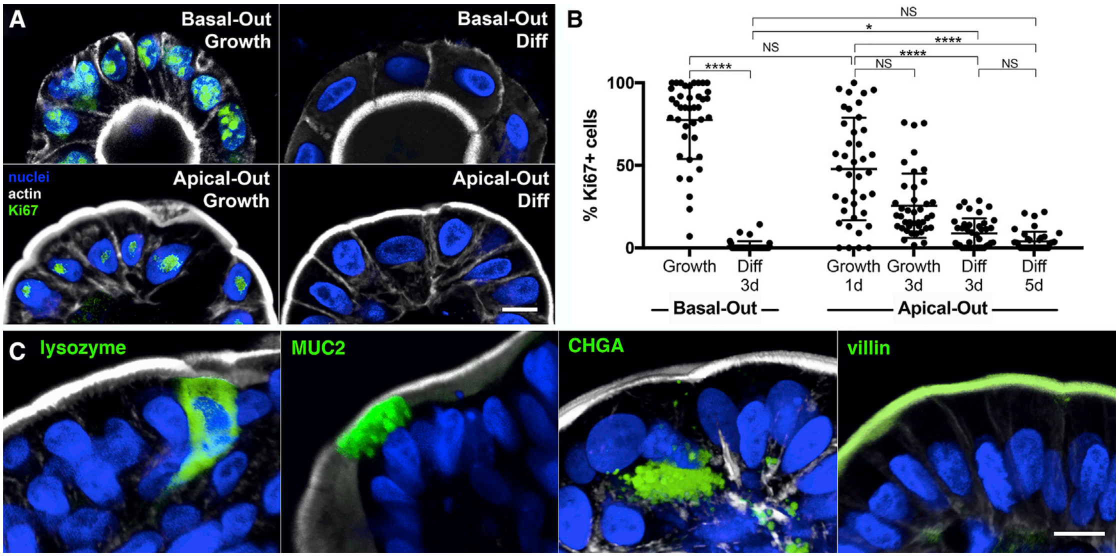

Figure 4. Apical-Out Enteroids Proliferate and Differentiate

(A) Basal-out enteroids and apical-out enteroids have proliferating cells when cultured in growth media (Growth), but not in differentiation media (Diff).

(B) Quantification of proliferation marker Ki67 shows that both basal-out and apical-out enteroid cells proliferate less in differentiation media (Diff) than in growth media (Growth). The percentage of proliferating cells in apical-out enteroids in growth media decreases over time. Data represented are mean ± SD; n = 40 enteroids; *$p < 0.05$; ****$p < 0.0001$; NS, not significant using the Kruskal-Wallis test with Dunn's multiple comparison test.

(C) Markers for different epithelial cell types (lysozyme for Paneth cells, MUC2 for goblet cells, CHGA for entero-endocrine cells, and villin for enterocytes) are expressed in apical-out enteroids.

Nuclei in blue and actin in white are shown. All scale bars are 10 μm. See also Figures S5 and S6.

diffusion assay (Figure 3) and found that infection of apical-out enteroids with *S.* Typhimurium results in decreased barrier integrity compared to uninfected enteroids (Figure S7).

S. Typhimurium invades host cells using a type 3 secretion system (T3SS), which injects effectors that induce host cell cytoskeletal rearrangement into actin "ruffles" that promote bacterial uptake (Galán, 2001). This mechanism has been well characterized using unpolarized or transformed epithelial cell lines, such as HeLa (Giannella et al., 1973), Caco-2 (Knodler et al., 2010), and T84 cells (Bishop et al., 2008). *S.* Typhimurium can induce actin ruffles and invade enterocytes in murine intestines (Knodler et al., 2010; Sellin et al., 2014) and ligated bovine ileal loops (Santos et al., 2002), but the mechanism of invasion of polarized primary human IECs has not been shown. We asked whether *S.* Typhimurium can induce actin ruffles to invade the apical surfaces of human enteroids and how this contrasts with bacteria's interactions with the basolateral surfaces. Apical-out enteroids or basal-out enteroids were infected with *S.* Typhimurium expressing mCherry (*S.* Typhimurium-mCherry) for 1 h and then fixed and stained for nuclei and actin. Confocal microscopy of infected apical-out enteroids showed that the bacteria can indeed induce actin ruffles to invade the human IECs (Figure 6A; Video S2). Comparison of *S.* Typhimurium infection in apical-out and basal-out enteroids revealed that the bacteria preferentially invade apical surfaces. In apical-out enteroids, a mean of 12.7 actin ruffles and 41.8 invading bacteria were observed per enteroid, and only 1.7 actin ruffles and 13.1 invading bacteria were observed per basal-out enteroid (Figures 6B–6D).

Mixed-polarity enteroids, in which only part of the enteroid has reversed polarity and thus contains both basal-out and apical-out regions, provide the opportunity to examine polarity-specific phenotypes within a single enteroid. These mixed polarity enteroids occur when polarity reversal is still in progress and has not been completed and also when enteroids with different polarities fuse together. We examined *S.* Typhimurium invasion of mixed polarity enteroids and observed that the bacteria invade the exposed apical surfaces, but not basolateral surfaces (Figure 6E, green arrows). This result confirms that *S.* Typhimurium preferentially invades the apical intestinal epithelial surfaces.

After invasion of the intestinal epithelium, *S*. Typhimurium replicates intracellularly and then induces extrusion of infected cells into the lumen. This process of infected cell extrusion has been documented in polarized Caco-2 colon carcinoma cells and in the murine intestine (Knodler et al., 2010). To determine whether human intestinal enteroids also support *S*. Typhimurium epithelial exit within extruding cells, we examined apical-out enteroids that had been infected with *S.* Typhimurium for 6 hours. Gentamicin was added to the media after the first hour to kill extracellular bacteria and prevent later invasion events. Using confocal microscopy, we show that there are bacteria located both within actively extruding epithelial cells (Figure 6F) and within fully extruded epithelial cells (Figure 6G).

Infection of Apical-Out Enteroids with *L. monocytogenes*

In contrast to *S.* Typhimurium's preference for apical infection, many enteric pathogens are known to utilize basolateral

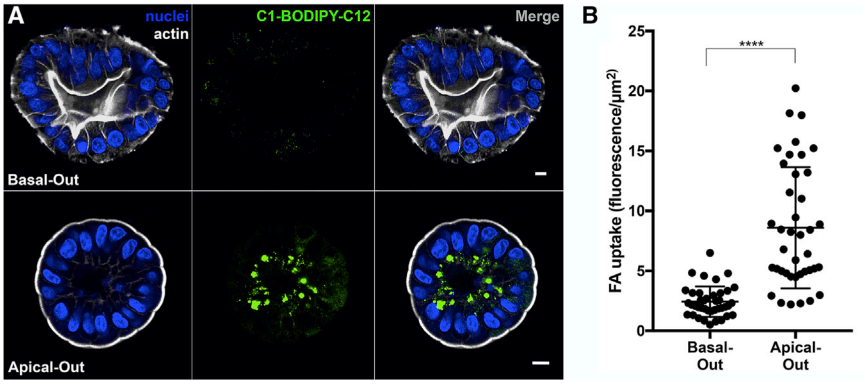

Figure 5. Apical Absorption of Fatty Acids in Enteroids

(A) Apical-out enteroids (bottom), but not basal-out enteroids (top), take up fluorescent fatty acid analog C1-BODIPY-C12 added to the extracellular media. Nuclei in blue and actin in white are shown. Scale bars are 10 μm.

(B) Quantification of fatty acid analog (FA) uptake in apical-out and basal-out enteroids.

Data represented are mean ± SD; n = 40 enteroids; $p < 0.0001$ using the Mann-Whitney U test.

receptors for invasion despite the lack of these receptors in the luminal surface. One example is *Listeria monocytogenes*, another bacterial pathogen that causes gastroenteritis and invasive disease in immunocompromised individuals. The host receptors for *L. monocytogenes* invasion are basolateral proteins E-cadherin and c-Met, and the pathogen has been described to enter epithelial cells more efficiently when it can access basolateral epithelial surfaces (Hamon et al., 2006). We have previously reported in polarized MDCK monolayers, in rabbit intestinal loop infections (Pentecost et al., 2006), and in a murine model of oral infection (Pentecost et al., 2010) that *L. monocytogenes* entry at the apical epithelial surface is restricted to sites of cell extrusion where cell polarity is transiently disrupted. To determine whether *L. monocytogenes* targets primary human IECs in this same manner, human enteroids were infected with *L. monocytogenes* expressing GFP (*L. monocytogenes*-GFP) and analyzed using confocal microscopy. After 15 minutes of infection, *L. monocytogenes* attached robustly to basal-out enteroids (60 bacteria per enteroid) and poorly to apical-out enteroids (4 bacteria per enteroid; Figures 7A and 7B). In mixed polarity enteroids, *L. monocytogenes* only attached to regions where basolateral surfaces were exposed (Figure 7C, red arrows). These results support previous findings that *L. monocytogenes* binds basolateral receptors. In the infected apical-out human enteroids, we observed that *L. monocytogenes* bacteria adhere almost exclusively at sites of extruding cells (Figure 7D). Thus, the apical-out human enteroid infection model recapitulates *L. monocytogenes* preferential targeting of sites of cell extrusion.

After initial attachment, *L. monocytogenes* enters host cells by endocytosis and escapes out of the vacuole. Once *L. monocytogenes* enters the host cell cytosol, the bacterium recruits and polymerizes host cell actin into actin "comet tails" to move within the cytosol and to form protrusions to invade adjacent epithelial cells (Hamon et al., 2006). To document that these processes occur in human enteroids, we examined apical-out enteroids infected with *L. monocytogenes*-GFP for 6 h. Gentamicin was added to the media after 1 h of infection to prevent later invasion events. Using confocal microscopy, we visualized intracellular bacteria that escaped the vacuole and recruited actin, as well as bacteria with polar polymerization of actin comet tails for intracellular motility (Figure 7E). We also documented bacteria with actin comet tails forming protrusions to invade neighboring cells (Figure 7F). *L. monocytogenes* was observed spreading locally into multiple neighboring enteroid cells through cell-to-cell spread after initial entry (Figure 7G). Interestingly, we discovered *L. monocytogenes* within actively extruding (Figure 7H) and fully extruded enteroid cells (Figure 7I).

DISCUSSION

In this work, we describe the development of a reversed-polarity apical-out human enteroid model for the gastrointestinal epithelium. This system facilitates access to the apical enteroid surface while possessing the benefits associated with the original Matrigel- or BME-embedded basal-out human enteroid method: the ability to generate lines from different patient biopsies that can also be genetically modified (Miyoshi and Stappenbeck, 2013), the development of polarity and tight junction function, and the presence of proliferative cells and differentiated IEC lineages. Apical-out enteroids differ from basal-out enteroids in several ways. First, apical-out enteroids have an outward-facing apical surface, which is easily accessible to experimental agents added to the culture media. Second, in contrast to basal-out enteroids that accumulate cellular debris inside the lumen, apical-out enteroids release mucus and extruded cells outward into the culture media, where they can be removed. Third, apical-out enteroids are suspended in media rather than embedded within BME, thus improving access to the enteroid surface because the BME scaffold can act as a diffusion barrier.

With BME-embedded basal-out human enteroids, microinjection is required to deliver experimental agents to the apical surface of the epithelium. This can be a technically challenging method because it requires growth of enteroids to large diameters to accommodate microinjection and can lead to spillage or contamination of the microinjected substances into the basolateral space. Moreover, normalizing exposure of microinjected experimental agents is difficult due to the small volumes, variability in enteroid size, and interactions with accumulated lumenal contents. To address these issues, another method to access the apical enteroid surface is to produce a polarized monolayer by seeding dissociated enteroid cells onto a Transwell permeable support. The Transwell system enables independent access to the apical and basolateral surfaces. However, producing a single monolayer requires many cells (typically

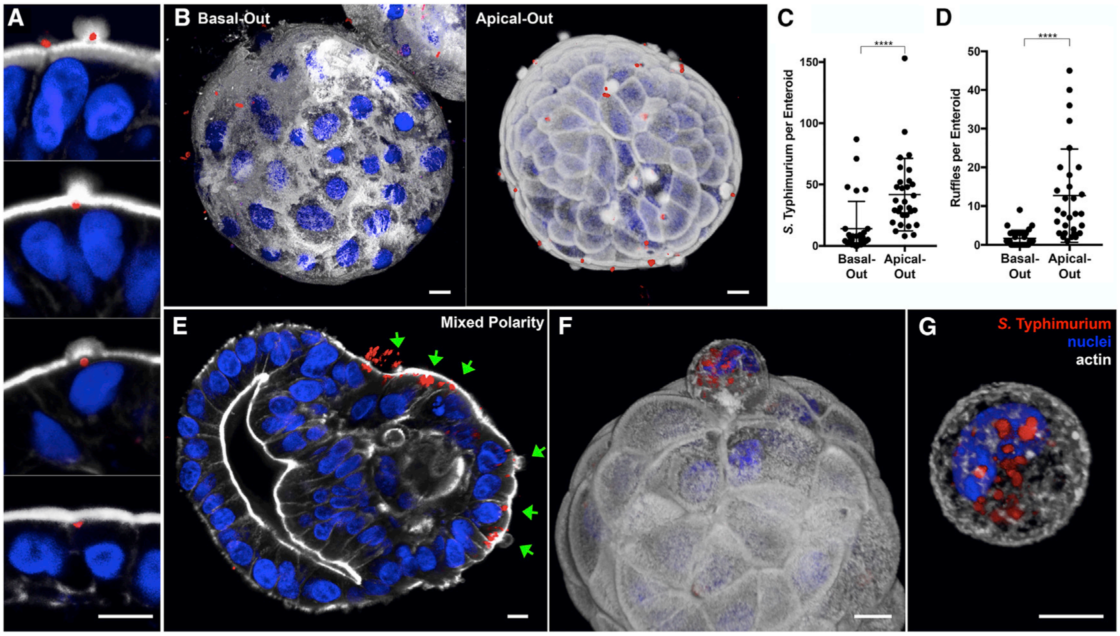

Figure 6. *S.* Typhimurium Infection of Human Enteroids

(A) *S.* Typhimurium-mCherry (red) at different stages of invasion of apical-out enteroids.

(B) 3D confocal reconstructions of basal-out enteroids and apical-out enteroids infected with *S.* Typhimurium-mCherry for 1 hour.

(C and D) Number of (C) invading bacteria and (D) actin ruffles per enteroid after 1 h of infection with *S.* Typhimurium-mCherry. Data represented are mean ± SD; n = 30 enteroids; p < 0.0001 using the Mann-Whitney U test.

(E) *S.* Typhimurium-mCherry selectively invades the exposed apical surface (green arrows) of a mixed polarity enteroid.

(F and G) 3D confocal reconstructions of *S.* Typhimurium-mCherry (F) within an epithelial cell in the process of extruding from the apical enteroid surface or (G) within a fully extruded cell after 6 h of infection.

Nuclei in blue and actin in white are shown. All scale bars are 10 μm. See also Figure S7 and Video S2.

pools of 1–3 wells of enteroids) and several days of monolayer maturation. Our method of generating apical-out enteroids shares some features of both of the prior methods. Similar to 2D monolayer and BME-embedded enteroid cultures, apical-out enteroids can be analyzed using a variety of experimental methods. Here, we demonstrate that our model is compatible with microscopy techniques for the evaluation of epithelial barrier integrity or nutrient uptake, flow cytometry for cell viability measurements, and qRT-PCR for transcriptional expression analysis. While it is difficult to examine 2D cultures by microscopy without disassembly of the Transwell system, BME-embedded enteroids and apical-out enteroids can both be easily monitored through live-cell imaging. However, apical-out enteroids provide an advantage over basal-out enteroids because the everted apical surface eliminates the need for microinjection. This enables normalized exposure to experimental substances, like with 2D enteroid monolayers. One advantage of apical-out enteroids over enteroid monolayers is that they form and develop proper polarity within hours of transfer to suspension culture. Experiments can be performed using small apical-out enteroids, thus saving growth time and reagents. Furthermore, because apical-out enteroids are in suspension, a single well can be separated into multiple wells to facilitate a larger number of experimental conditions.

Our results indicate that the enteroid polarity reversal is mediated by the removal of ECM components, because the frequency of polarity reversal decreased with increasing concentrations of BME in the suspension cultures. Furthermore, we found that a receptor for ECM proteins, β1 integrin, plays an important role in regulating enteroid polarity, because treatment with an β1 integrin function-blocking antibody reversed the polarity of BME-embedded enteroids to an apical-out orientation, despite that the enteroids were still embedded in the matrix. Our findings are consistent with previous reports that β1 integrin signaling controls epithelial polarity in MDCK spheroids (Ojakian and Schwimmer, 1994; Yu et al., 2005). Based on previous studies using MDCK spheroids (Wang et al., 1990), we suspected that epithelial polarity reversal may occur as a result of migration of proteins and organelles from one pole to the other. However, the time-lapse microscopy analysis of polarity reversal in immobilized apical-out enteroids revealed that the spheroids undergo a dramatic morphological rearrangement in which the internal apical surface everts as the basolateral surfaces become internalized. Confocal micrographs of suspended enteroids at different stages of polarity reversal matched the morphological changes observed in the time-lapse microscopy experiment for immobilized apical-out enteroids. Instead of the protein and organelle migration phenotype reported for MDCK spheroids, the enteroid polarity reversal process more closely resembles previous reports of epithelial polarity regulation via eversion in porcine thyroid epithelial sheets (Graebert et al., 1997). It is possible that there are also other mechanisms, such as

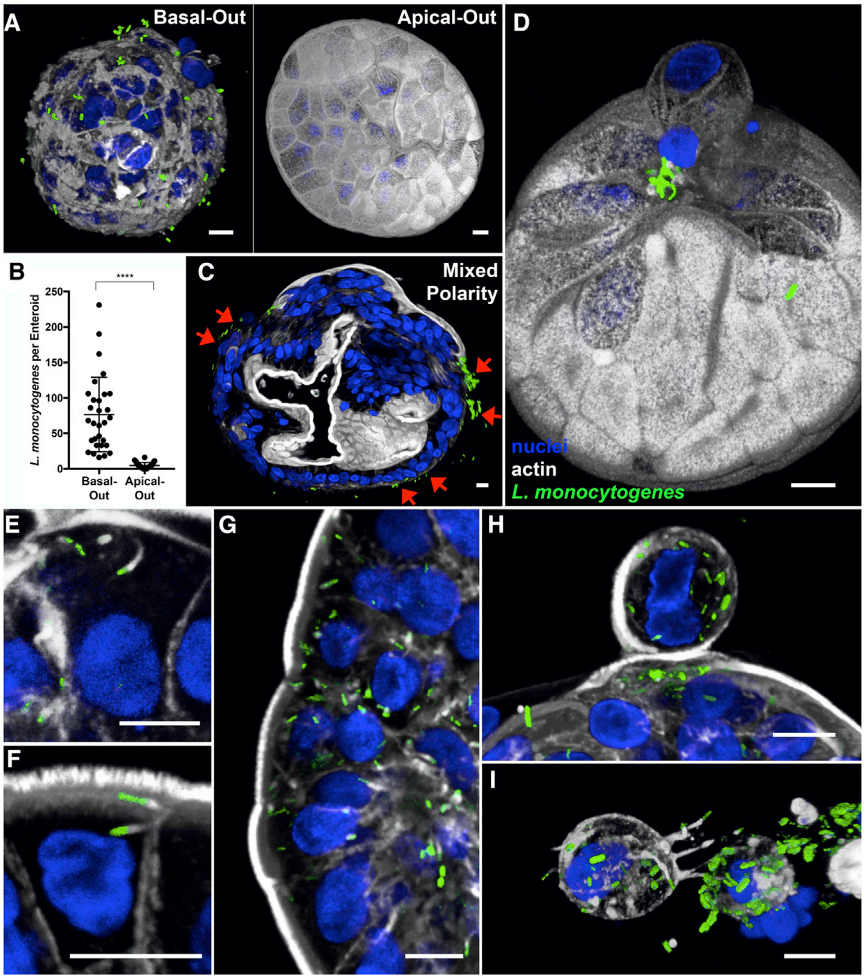

Figure 7. *L. monocytogenes* Infection of Human Enteroids

(A) 3D confocal reconstruction of *L. monocytogenes*-GFP (green) attached to basal-out enteroids or apical-out enteroids after 15 min of infection.

(B) Quantification of bacteria associated with basal-out or apical-out enteroids after 15 min of infection. Data represented are mean ± SD; n = 30 enteroids; $p < 0.0001$ using the Mann-Whitney U test.

(C) *L. monocytogenes*-GFP selectively attaches to basal-out regions of a mixed polarity enteroid after 15 minutes of infection (red arrows).

(D) 3D confocal reconstruction of a 15 minute infection shows that *L. monocytogenes*-GFP selectively attaches to apical-out enteroids at sites of cell extrusion.

(E and F) After 6 h of infection, (E) intracellular *L. monocytogenes*-GFP are seen recruiting and polymerizing actin (white) into comet tails to move within the cytosol and (F) to invade neighboring cells.

(G) Intracellular *L. monocytogenes*-GFP in patches of enteroid cells due to cell-to-cell spread.

(H and I) In apical-out enteroids infected with *L. monocytogenes*-GFP for 6 h, bacteria exit the epithelium within (H) actively extruding cells or (I) completely extruded cells.

Nuclei in blue and actin in white are shown. All scale bars are 10 μm.

responses to mechanical forces, that contribute to the regulation of enteroid eversion and polarity, and the method described in this study provides a platform to further investigate coordinated epithelial morphogenesis.

The apical-out human enteroid model recapitulates important complexities and functions of the native intestinal epithelium. Similar to basal-out human enteroids, apical-out enteroids differentiate to the various IEC cell lineages upon removal of Wnt and R-spondin growth factors from the cultures. Interestingly, compared to basal-out enteroids, we found apical-out enteroids skewed more toward differentiation into absorptive enterocytes. As reported for basal-out enteroids (Basak et al., 2017; Luu et al., 2018), optimization of media conditions for apical-out enteroids will be useful to skew differentiation toward IEC lineages of interest. We demonstrated that the apical-out enteroids can absorb a fluorescent fatty acid analog through the apical surface and incorporate it into lipid droplets, which are then trafficked to the basal region of the cell. Basal-out enteroids do not efficiently absorb the fluorescent fatty acid analog. The apical-specific uptake of fatty acids is consistent with studies in polarized Caco-2 cells (Trotter and Storch, 1991). This model can be implemented for more extensive studies of enterocyte physiology. Studying apical-out enteroids from different donors could provide insight to intestinal phenotypes in health and disease, demographic and individual variability, as well as epithelial responses to and uptake of drugs and therapeutics.

We developed this model in particular to facilitate studies of host-pathogen interactions with the human gastrointestinal epithelium. Human enteroids have emerged as a promising system to study both commensal and pathogenic microbes, many of which have previously been difficult to study because they are human specific or human restricted. Apical-out human enteroids are readily infected by simply adding microbes of interest to the culture media. Here, we used the suspended enteroid culture system to compare pathogen interactions with apical and basolateral epithelial surfaces. We first studied enteroid infection by *S.* Typhimurium, whose host cell invasion mechanisms have been well characterized in transformed epithelial cell lines, but not in polarized primary human IECs. We found that, in human enteroids, *S.* Typhimurium invades and induces actin ruffles more efficiently in apical surfaces than in basolateral surfaces. These results contrast with previous publications using polarized monolayers of epithelial cell lines, in which *S.* Typhimurium invaded the apical and basolateral surfaces at equal frequencies (Bishop et al., 2008; Criss and Casanova, 2003). One possibility is that the non-human (MDCK; Criss and Casanova, 2003) and transformed (T84; Bishop et al., 2008) cell lines used in these studies differ from human IECs. In agreement with our findings,

a recent study showed that, in polarized Caco-2 colon carcinoma cells, depletion of the apical protein villin resulted in reduced actin ruffle size and decreased *S.* Typhimurium invasion (Lhocine et al., 2015). The importance of villin, which is only expressed on apical epithelial cell surfaces, may contribute to the bacteria's preference for invasion of apical enteroid surfaces. Most studies of *S.* Typhimurium infection in animals have shown preferential invasion only of microfold cells (M cells); however, in these models, *S.* Typhimurium causes systemic infection that resembles typhoid fever. In humans, *S.* Typhimurium rarely results in systemic infection and instead causes acute inflammatory gastroenteritis, suggesting a more diffuse process of local epithelial invasion like that visualized in our apical-out enteroid model.

The apical infection preference of *S.* Typhimurium is juxtaposed by the basolateral preference of *L. monocytogenes* in human enteroids. The basolateral enteroid preference is consistent with reported basolateral receptors for *L. monocytogenes*, E-cadherin, and c-Met (Hamon et al., 2006). It has been a puzzle why *L. monocytogenes* and other enteric pathogens have evolved strategies to bind to basolateral receptors, which are not readily accessible from the intestinal lumen. *L. monocytogenes* has been postulated to enter the epithelium through M cells (Jensen et al., 1998), where basolateral proteins are apically expressed. We proposed that the regions of cell extrusion at the villus tips are an entry site for *L. monocytogenes* during human intestinal infection *in vivo*. We previously determined that *L. monocytogenes* can enter the polarized apical epithelium at sites of cell extrusion in polarized MDCK monolayers (Pentecost et al., 2006). We also had observed that, in a rabbit intestinal loop model of infection, *L. monocytogenes* invaded at sites of extruding cells at the villus tips, where cells are shed during normal epithelial regeneration (Pentecost et al., 2006). This same preference for the tips of the intestinal villi was observed in mice infected with murine-adapted *L. monocytogenes* (Pentecost et al., 2010). At areas of cell extrusion, the bacteria's basolateral receptor E-cadherin was apically exposed on the extruding cell and neighboring cells during epithelial remodeling. Here, we show that *L. monocytogenes* also targets sites of cell extrusion to enter the apical epithelium in human enteroids, thus confirming the previous reports in non-human models. The apical-out human enteroid infection model highlights the importance of cell polarity and, in particular, sites of cell extrusion for pathogens with basolateral receptors. Numerous enteric pathogens have basolateral receptors for invasion, including *Shigella flexneri* and rotavirus. Our model is amenable to test whether these pathogens can also find their receptors at sites of cell extrusion, where basolateral proteins are apically exposed.

We speculate that regions of cell extrusion would be a favorable infection site for several reasons. First, during and after an extrusion event, these regions are actively remodeling and endocytosing membrane components. These areas allow pathogen entry through endocytosis (Pentecost et al., 2010) without causing epithelial injury, potentially avoiding immune responses associated with tissue damage. Second, the extrusion of infected cells at the villus tips and neighboring cells, in which pathogen replication has occurred, could facilitate pathogen persistence and transmission by shedding into the environment. *S.* Typhimurium has been previously reported to induce cell extrusion after intracellular replication within Caco-2 colon carcinoma cells and murine IECs (Knodler et al., 2010). We observed that *S.* Typhimurium can exit apical-out human enteroids inside extruding cells, thus recapitulating findings from mouse and transformed cell culture models.

Although we previously suggested that *L. monocytogenes* exits the epithelium via extruding cells at the tips of the intestinal villi (Pentecost et al., 2006), this process was difficult to visualize due to the rapid loss of extruded cells in the lumen. Our findings using the apical-out human enteroid model allowed us to visualize this mechanism of *L. monocytogenes* exit from the epithelium to promote shedding and dissemination. The apical-out enteroid model will enable further studies about the specific roles and mechanisms of cell extrusion during infection by *S.* Typhimurium, *L. monocytogenes*, and potentially other gastrointestinal pathogens. In contrast to other modes of pathogen egress from a host cell, such as cell lysis, cell extrusion is a natural method of cell expulsion that pathogens could hijack to subvert immune activation. Pathogens could actively induce cell extrusion, as has been reported for *S.* Typhimurium (Knodler et al., 2010), or might passively "hitch a ride" in cells that extrude during normal epithelial cell turnover. The extruded cell might represent a pathogen's "escape pod" that provides a nutrient source and temporary protection from antibody recognition or the harsh lumenal environment and ultimately facilitates re-infection of more distal regions of the gut and shedding into the environment.

Overall, apical-out human enteroids are a relevant and accessible model for the human gastrointestinal epithelium. This experimental system has enabled the recapitulation and advancement of our understanding of gastrointestinal infections. We believe that this model has the potential to facilitate future discoveries about gastrointestinal health and disease.

STAR★METHODS

Detailed methods are provided in the online version of this paper and include the following:

- KEY RESOURCES TABLE
- CONTACT FOR REAGENT AND RESOURCE SHARING
- EXPERIMENTAL MODEL AND SUBJECT DETAILS
  - Human enteroid cultivation
  - Mouse enteroid cultivation
  - Enteroid suspension culture
  - Bacterial strains
- METHOD DETAILS
  - Cell viability measurements
  - Confocal microscopy
  - Antibody treatments
  - qRT-PCR
  - Epithelial barrier integrity
  - Fatty acid absorption
  - Time lapse microscopy of immobilized apical-out enteroids

- ○ Enteroid infections
- QUANTIFICATION AND STATISTICAL ANALYSIS

SUPPLEMENTAL INFORMATION

Supplemental Information can be found with this article online at https://doi.org/10.1016/j.celrep.2019.01.108.

ACKNOWLEDGMENTS

We thank Connie Fung and Rachel Cooper for assistance deriving murine enteroids, Liliana Massis for help analyzing flow cytometry data, and David Schneider for access to the Accuri Cytometer. This research was supported by the Stanford Child Health Research Institute Postdoctoral Award (to J.Y.C.), NIH T32AI007328-29 (support to J.Y.C.), NIH U19AI116484-01 (to M.R.A., D.M.M., and C.J.K.), and Bill and Melinda Gates Foundation OPP1113682 (Stanford CHSI Pilot Grant to M.R.A. and D.M.M).

AUTHOR CONTRIBUTIONS

J.Y.C., D.M.M., and M.R.A. conceived the study. J.Y.C., D.M.M., and M.R.A. designed the experiments. J.Y.C. performed most of the experiments. J.Y.C. and M.M.-C. performed and analyzed the time-lapse experiments. A.T.M., X.L., and C.J.K. generated the enteroids and provided reagents for this study. J.Y.C., D.M.M., and M.R.A. analyzed the data and wrote the manuscript. All authors provided comments on the manuscript.

DECLARATION OF INTERESTS

C.J.K. is a founder, scientific advisory board member, and shareholder of both TOMA Biosciences and Surrozen, Inc. C.J.K. is a consultant for BioTechne.

Received: June 26, 2018
Revised: November 20, 2018
Accepted: January 30, 2019
Published: February 26, 2019

REFERENCES

Bartfeld, S., and Clevers, H. (2015). Organoids as model for infectious diseases: culture of human and murine stomach organoids and microinjection of *Helicobacter pylori*. J. Vis. Exp. *105*, e53359.

Bartfeld, S., Bayram, T., van de Wetering, M., Huch, M., Begthel, H., Kujala, P., Vries, R., Peters, P.J., and Clevers, H. (2015). In vitro expansion of human gastric epithelial stem cells and their responses to bacterial infection. Gastroenterology *148*, 126–136.e6.

Basak, O., Beumer, J., Wiebrands, K., Seno, H., van Oudenaarden, A., and Clevers, H. (2017). Induced quiescence of Lgr5+ stem cells in intestinal organoids enables differentiation of hormone-producing enteroendocrine cells. Cell Stem Cell *20*, 177–190.e4.

Bishop, A., House, D., Perkins, T., Baker, S., Kingsley, R.A., and Dougan, G. (2008). Interaction of *Salmonella enterica* serovar Typhi with cultured epithelial cells: roles of surface structures in adhesion and invasion. Microbiology *154*, 1914–1926.

Coburn, B., Grassl, G.A., and Finlay, B.B. (2007). Salmonella, the host and disease: a brief review. Immunol. Cell Biol. *85*, 112–118.

Criss, A.K., and Casanova, J.E. (2003). Coordinate regulation of *Salmonella enterica* serovar Typhimurium invasion of epithelial cells by the Arp2/3 complex and Rho GTPases. Infect. Immun. *71*, 2885–2891.

Drecktrah, D., Levine-Wilkinson, S., Dam, T., Winfree, S., Knodler, L.A., Schroer, T.A., and Steele-Mortimer, O. (2008). Dynamic behavior of Salmonella-induced membrane tubules in epithelial cells. Traffic *9*, 2117–2129.

Ettayebi, K., Crawford, S.E., Murakami, K., Broughman, J.R., Karandikar, U., Tenge, V.R., Neill, F.H., Blutt, S.E., Zeng, X.-L., Qu, L., et al. (2016). Replication of human noroviruses in stem cell-derived human enteroids. Science *353*, 1387–1393.

Finlay, B.B., and Falkow, S. (1990). *Salmonella* interactions with polarized human intestinal Caco-2 epithelial cells. J. Infect. Dis. *162*, 1096–1106.

Galán, J.E. (2001). *Salmonella* interactions with host cells: type III secretion at work. Annu. Rev. Cell Dev. Biol. *17*, 53–86.

Giannella, R.A., Washington, O., Gemski, P., and Formal, S.B. (1973). Invasion of HeLa cells by *Salmonella* typhimurium: a model for study of invasiveness of Salmonella. J. Infect. Dis. *128*, 69–75.

Graebert, K.S., Bauch, H., Neumüller, W., Brix, K., and Herzog, V. (1997). Epithelial folding in vitro: studies on the cellular mechanism underlying evagination of thyrocyte monolayers. Exp. Cell Res. *231*, 214–225.

Hamon, M., Bierne, H., and Cossart, P. (2006). *Listeria monocytogenes*: a multifaceted model. Nat. Rev. Microbiol. *4*, 423–434.

Hecht, G. (1995). Bugs and barriers: enteric pathogens exploit yet another epithelial function. Physiology (Bethesda) *10*, 160–166.

Holly, M.K., and Smith, J.G. (2018). Adenovirus infection of human enteroids reveals interferon sensitivity and preferential infection of goblet cells. J. Virol. *92*, e00250-18.

Jensen, V.B., Harty, J.T., and Jones, B.D. (1998). Interactions of the invasive pathogens Salmonella typhimurium, Listeria monocytogenes, and *Shigella flexneri* with M cells and murine Peyer's patches. Infect. Immun. *66*, 3758–3766.

Knodler, L.A., Vallance, B.A., Celli, J., Winfree, S., Hansen, B., Montero, M., and Steele-Mortimer, O. (2010). Dissemination of invasive *Salmonella* via bacterial-induced extrusion of mucosal epithelia. Proc. Natl. Acad. Sci. USA *107*, 17733–17738.

Lhocine, N., Arena, E.T., Bomme, P., Ubelmann, F., Prévost, M.-C., Robine, S., and Sansonetti, P.J. (2015). Apical invasion of intestinal epithelial cells by *Salmonella* typhimurium requires villin to remodel the brush border actin cytoskeleton. Cell Host Microbe *17*, 164–177.

Luu, L., Matthews, Z.J., Armstrong, S.D., Powell, P.P., Wileman, T., Wastling, J.M., and Coombes, J.L. (2018). Proteomic profiling of enteroid cultures skewed toward development of specific epithelial lineages. Proteomics *18*, e1800132.

Miyoshi, H., and Stappenbeck, T.S. (2013). In vitro expansion and genetic modification of gastrointestinal stem cells in spheroid culture. Nat. Protoc. *8*, 2471–2482.

Ojakian, G.K., and Schwimmer, R. (1994). Regulation of epithelial cell surface polarity reversal by beta 1 integrins. J. Cell Sci. *107*, 561–576.

Pagano, R.E., and Sleight, R.G. (1985). Defining lipid transport pathways in animal cells. Science *229*, 1051–1057.

Pentecost, M., Otto, G., Theriot, J.A., and Amieva, M.R. (2006). *Listeria monocytogenes* invades the epithelial junctions at sites of cell extrusion. PLoS Pathog. *2*, e3.

Pentecost, M., Kumaran, J., Ghosh, P., and Amieva, M.R. (2010). *Listeria monocytogenes* internalin B activates junctional endocytosis to accelerate intestinal invasion. PLoS Pathog. *6*, e1000900.

Rajan, A., Vela, L., Zeng, X.-L., Yu, X., Shroyer, N., Blutt, S.E., Poole, N.M., Carlin, L.G., Nataro, J.P., Estes, M.K., et al. (2018). Novel segment- and host-specific patterns of Enteroaggregative *Escherichia coli* adherence to human intestinal enteroids. MBio *9*, e02419-17.

Santos, R.L., Zhang, S., Tsolis, R.M., Bäumler, A.J., and Adams, L.G. (2002). Morphologic and molecular characterization of Salmonella typhimurium infection in neonatal calves. Vet. Pathol. *39*, 200–215.

Sato, T., Vries, R.G., Snippert, H.J., van de Wetering, M., Barker, N., Stange, D.E., van Es, J.H., Abo, A., Kujala, P., Peters, P.J., and Clevers, H. (2009). Single Lgr5 stem cells build crypt-villus structures in vitro without a mesenchymal niche. Nature *459*, 262–265.

Sato, T., Stange, D.E., Ferrante, M., Vries, R.G.J., Van Es, J.H., Van den Brink, S., Van Houdt, W.J., Pronk, A., Van Gorp, J., Siersema, P.D., and Clevers, H. (2011). Long-term expansion of epithelial organoids from human colon,

adenoma, adenocarcinoma, and Barrett's epithelium. Gastroenterology *141*, 1762–1772.

Saxena, K., Blutt, S.E., Ettayebi, K., Zeng, X.-L., Broughman, J.R., Crawford, S.E., Karandikar, U.C., Sastri, N.P., Conner, M.E., Opekun, A.R., et al. (2015). Human intestinal enteroids: a new model to study human rotavirus infection, host restriction, and pathophysiology. J. Virol. *90*, 43–56.

Sellin, M.E., Müller, A.A., Felmy, B., Dolowschiak, T., Diard, M., Tardivel, A., Maslowski, K.M., and Hardt, W.-D. (2014). Epithelium-intrinsic NAIP/NLRC4 inflammasome drives infected enterocyte expulsion to restrict *Salmonella* replication in the intestinal mucosa. Cell Host Microbe *16*, 237–248.

Spence, J.R., Mayhew, C.N., Rankin, S.A., Kuhar, M.F., Vallance, J.E., Tolle, K., Hoskins, E.E., Kalinichenko, V.V., Wells, S.I., Zorn, A.M., et al. (2011). Directed differentiation of human pluripotent stem cells into intestinal tissue *in vitro*. Nature *470*, 105–109.

Trotter, P.J., and Storch, J. (1991). Fatty acid uptake and metabolism in a human intestinal cell line (Caco-2): comparison of apical and basolateral incubation. J. Lipid Res. *32*, 293–304.

VanDussen, K.L., Marinshaw, J.M., Shaikh, N., Miyoshi, H., Moon, C., Tarr, P.I., Ciorba, M.A., and Stappenbeck, T.S. (2015). Development of an enhanced human gastrointestinal epithelial culture system to facilitate patient-based assays. Gut *64*, 911–920.

Wang, A.Z., Ojakian, G.K., and Nelson, W.J. (1990). Steps in the morphogenesis of a polarized epithelium. II. Disassembly and assembly of plasma membrane domains during reversal of epithelial cell polarity in multicellular epithelial (MDCK) cysts. J. Cell Sci. *95*, 153–165.

Wang, T.Y., Liu, M., Portincasa, P., and Wang, D.Q.-H. (2013). New insights into the molecular mechanism of intestinal fatty acid absorption. Eur. J. Clin. Invest. *43*, 1203–1223.

Yu, W., Datta, A., Leroy, P., O'Brien, L.E., Mak, G., Jou, T.-S., Matlin, K.S., Mostov, K.E., and Zegers, M.M.P. (2005). Beta1-integrin orients epithelial polarity via Rac1 and laminin. Mol. Biol. Cell *16*, 433–445.

Yurchenco, P.D., Tsilibary, E.C., Charonis, A.S., and Furthmayr, H. (1985). Laminin polymerization in vitro. Evidence for a two-step assembly with domain specificity. J. Biol. Chem. *260*, 7636–7644.

Zhang, Y.-G., Wu, S., Xia, Y., and Sun, J. (2014). *Salmonella*-infected crypt-derived intestinal organoid culture system for host-bacterial interactions. Physiol. Rep. *2*, e12147.

STAR★METHODS

KEY RESOURCES TABLE

REAGENT or RESOURCE	SOURCE	IDENTIFIER
Antibodies		
Mouse anti-ZO-1 clone 1A12	Invitrogen	Cat# 33-9100; RRID:AB_2533147
Rabbit anti-β-catenin clone H-102	SCBT	Cat# sc-7199; RRID:AB_634603
Rabbit anti-Lysozyme	Dako	Cat# A0099; RRID:AB_2341230
Rabbit anti-MUC2 clone H-300	SCBT	Cat# sc-15334; RRID:AB_2146667
Mouse anti-Villin clone 1D2C3	SCBT	Cat# sc-58897; RRID:AB_2304475
Rabbit anti-Ki67 clone SP6	Invitrogen	Cat# MA5-14520; RRID:AB_10979488
Rat anti-β1 integrin neutralizing antibody clone AIIB2 supernatant	Developmental Studies Hybridoma Bank	Cat# AIIB2 s; RRID:AB_528306
Mouse anti-LAMP-1 clone H4A3 supernatant	Developmental Studies Hybridoma Bank	Cat# H4A3 s; AB_528126
Bacterial and Virus Strains		
Salmonella enterica Typhimurim SL1344 pFPV25-mCherry	(Drecktrah et al., 2008)	N/A
Listeria monocytogenes 10403s pMP74	(Pentecost et al., 2010)	N/A
Biological Samples		
Human small intestinal (ileal) tissue	Stanford Tissue Bank	N/A
Human colon tissue	Stanford Tissue Bank	N/A
Human gastric tissue	Stanford Tissue Bank	N/A
Chemicals, Peptides, and Recombinant Proteins		
Cultrex Reduced Growth Factor Basement Membrane Matrix (BME), Type II	BioTechne	Cat# 3533-001-02
WRN conditioned media	(Miyoshi and Stappenbeck, 2013)	N/A
Advanced DMEM/F12	ThermoFisher	Cat# 12634028
HEPES	ThermoFisher	Cat# 15630080
Glutamax	ThermoFisher	Cat# 35050061
N-acetyl-cysteine (NAC)	Sigma Aldrich	Cat# A7250
Nicotinamide (NIC)	Sigma Aldrich	Cat# N0636
SB202190	Sigma Aldrich	Cat# S7076
B-27 (w/o vitamin A)	ThermoFisher	Cat# 12587001
A83-01	BioTechne	Cat# 2939
Gastrin	Sigma Aldrich	Cat# G9145
EGF	Peprotech	Cat# AF-100-15
γ-Secretase Inhibitor IX (DAPT)	EMD Millipore	Cat# 565770
FGF10	Peprotech	Cat# 100-26
Y27632	Peprotech	Cat# 1293823
CHIR99021	R&D Systems	Cat# 4423/10
SYTOX Green	Invitrogen	Cat# S7020
FITC-dextran 4kDa	Sigma Aldrich	Cat# 46944
BODIPY 500/510 C1, C12	ThermoFisher	Cat# D3823
BSA, fatty acid free	Sigma Aldrich	Cat# A8806
Critical Commercial Assays		
RNeasy Plus Micro Kit	QIAGEN	Cat# 74034
SuperScript III First-Strand Synthesis System	Invitrogen	Cat# 18080051
FastStart Universal SYBR Green Master (Rox)	Roche	Cat# 4913914001

(*Continued on next page*)

Continued

REAGENT or RESOURCE	SOURCE	IDENTIFIER
Experimental Models: Organisms/Strains		
C57BL/6J mice	The Jackson Laboratory	N/A
Oligonucleotides		
qRT-PCR primer: GAPDH Fw: GACCTGCCGTCTAGAAAAACC	(VanDussen et al., 2015)	N/A
qRT-PCR primer: GAPDH Rev: GCTGTAGCCAAATTCGTTGTC	(VanDussen et al., 2015)	N/A
qRT-PCR primer: CHGA Fwd: AGAATTTACTGAAGGAGCTCCAAG	(VanDussen et al., 2015)	N/A
qRT-PCR primer: CHGA Rev: TCCTCTCTTTTCTCCATAACATCC	(VanDussen et al., 2015)	N/A
qRT-PCR primer: LYZ Fwd: GGTTACAACACACGAGCTACAAAC	(VanDussen et al., 2015)	N/A
qRT-PCR primer: LYZ Rev: AGTTACACTCCACAACCTTGAACA	(VanDussen et al., 2015)	N/A
qRT-PCR primer: MUC2 Fwd: AGGATCTGAAGAAGTGTGTCACTG	(VanDussen et al., 2015)	N/A
qRT-PCR primer: MUC2 Rev: TAATGGAACAGATGTTGAAGTGCT	(VanDussen et al., 2015)	N/A
qRT-PCR primer: SI Fwd: CTGCATTTGAAAGAGGACAGC	(VanDussen et al., 2015)	N/A
qRT-PCR primer: SI Rev: ACTCTGCTGTGGAAGTCCTGA	(VanDussen et al., 2015)	N/A
qRT-PCR primer: LGR5 Fwd: TATGCCTTTGGAAACCTCTC	(Bartfeld et al., 2015)	N/A
qRT-PCR primer: LGR5 Rev: CACCATTCAGAGTCAGTGTT	(Bartfeld et al., 2015)	N/A

CONTACT FOR REAGENT AND RESOURCE SHARING

Further information and requests for reagents may be directed to and will be fulfilled by the Lead Contact, Manuel R. Amieva (amieva@stanford.edu).

EXPERIMENTAL MODEL AND SUBJECT DETAILS

Human enteroid cultivation

Enteroids were derived as described in Sato et al. (2011). All human enteroids in this study were derived from small intestinal ileal tissue unless otherwise stated. De-identified human tissue samples were procured by the Stanford Tissue Bank with patient consent and approval by the Stanford University IRB. Samples were obtained from surgical gastrointestinal tissue without a particular targeted or planned enrollment. Age and sex information was not specifically collected for this study. Tissue samples were washed with cold PBS until the supernatant was clear. Tissue fragments were incubated in 2 mM EDTA (for intestinal samples) or 10 mM EDTA (for gastric samples) in cold chelation buffer (distilled water with 5.6 mM Na_2HPO_4, 8.0 mM KH_2PO_4, 96.2 mM NaCl, 1.6 mM KCl, 43.4 mM sucrose, 54.9 mM d-sorbitol, 0.5 mM DL-dithiothreitol) on ice for 30 minutes (for intestinal samples) or 3–5 hours (for gastric samples). The EDTA buffer was removed and tissue fragments were vigorously shaken in cold chelation buffer to isolate intestinal crypts or gastric glands. The tissue fragments were allowed to sink under normal gravity for 1 minute, and the supernatant was removed for inspection by inverted microscopy. This process was repeated until intact crypts/glands were visible by microscopy. The supernatants containing crypts/glands were collected in 15 mL Falcon tubes. Isolated crypts/glands were pelleted, washed with cold chelation buffer, and centrifuged at 200 x g for 3 minutes to separate from single cells. Isolated glands were embedded in Cultrex Reduced Growth Factor Basement Membrane Matrix, Type II (BME, which is equivalent to Matrigel) on ice and seeded into a 24-well tissue culture plate. BME was incubated for at least 10 minutes at 37°C to polymerize. Then 400-500 μL of growth media (Advanced Dulbecco's modified Eagle medium/F12, 1 mM HEPES, 1x Glutamax, 1x B27, 1 mM N-Acetyl-cysteine, and the following growth factors: 10 nM Gastrin, 50 ng/mL EGF, 10 mM Nicotinamide, 500 nM A83-01, 10 μM SB202190, 100ng/mL FGF10 (for gastric samples only) and 50% L-WRN-conditioned media (contains Wnt3a, R-spondin 3, and Noggin) (Miyoshi and Stappenbeck, 2013). 10 μM Y27632 and 250 nM CHIR99021 were also included in the media for the first 3-4 days after passage. Growth media was replaced every 2-4 days. Enteroid cultures were passaged every 1-2 weeks by digesting enteroids with TrypLE Express (ThermoFisher) in a 37°C water bath for 10 minutes. 1/10 volume FBS was added to inactivate TrypLE Express and cells were pelleted at 500 x g. Cells were re-embedded into fresh BME and plated in 24-well plates. For enteroid differentiation, growth media was replaced with an equal volume of differentiation media (modified from Saxena et al., 2015): Advanced Dulbecco's modified Eagle medium/F12, 10% FBS, 5 μM DAPT, 1x B-27, 1 mM N-acetyl-cysteine, 10 nM Gastrin, 50 ng/mL EGF, 100 ng/mL Noggin, 500 nM A83-01, and 10 μM Y27632) and incubated for at least 3 days. During growth, enteroids were monitored either by modulation contrast microscopy using a 10x or 40x objective on an CK2 inverted microscope (Olympus) with a XC-77 CCD Camera (Hamamatsu), or using a dissecting microscope (Bausch & Lomb) with a Samsung Galaxy S7 phone to capture images.

Mouse enteroid cultivation

Murine enteroids were derived from duodenal tissue from C57BL/6J mice that were ≥ 5 weeks of age (The Jackson Laboratory). All animal experiments were performed in accordance with NIH guidelines and with approval from the Institutional Animal Care and Use Committee of Stanford University. Enteroids were derived from tissue as described above for human enteroids.

Enteroid suspension culture

Enteroids were grown embedded in BME for 7-20 days with growth media. BME-embedded enteroids were dislodged with a sterile spatula and solubilized in 5 mM EDTA in PBS for 1 h at 4°C on a rotating platform. Enteroids were centrifuged at 200 x g for 3 min at 4°C and the supernatant was removed. The pellet was re-suspended in growth media or differentiation media in ultra-low attachment 24-well tissue culture plates (Corning Costar 3473). Suspended enteroids were incubated at 37°C with 5% CO_2 for 3 days prior to use unless otherwise stated. For experiments with BME-containing media, cold BME was titrated into ice cold growth media to prevent heterogeneous polymerization of BME into clumps.

Bacterial strains

Bacterial strains *Salmonella enterica* serovar Typhimurium SL1344 pFPV25-mCherry (*S.* Typhimurium-mCherry), *Listeria monocytogenes* 10403s pMP74 (*L. monocytogenes*-GFP) were used (Pentecost et al., 2010). For *S.* Typhimurium-mCherry, single colonies grown on LB agar plates with carbenicillin (50 μg/mL) were inoculated into LB broth with carbenicillin (50 μg/mL) and grown shaking overnight at 37°C. Overnight cultures were subcultured 1:20 into LB with 300 mM NaCl and grown standing at 37°C with 5% CO_2 for 2-3 h. For *L. monocytogenes*-GFP, single colonies were inoculated into BHI with chloramphenicol (8 μg/mL) and grown statically at room temperature overnight.

METHOD DETAILS

Cell viability measurements

Enteroids embedded in BME or in suspension culture with growth media for 1 day were harvested and dissociated with TrypLE Express for 30 minutes in a 37°C water bath. Enteroid cells were pelleted and re-suspended in 10 nM SYTOX Green Nucleic Acid Stain (Invitrogen) in PBS for 10 minutes on ice. Cells were analyzed on an Accuri Cytometer (BD Biosciences) to determine the frequency of SYTOX Green positive cells (dead cells) and analyzed using FlowJo software (FlowJo).

Confocal microscopy

Enteroids were fixed in 2% paraformaldehyde in 100 mM phosphate buffer (pH 7.4) for 30 minutes at room temperature, then washed 2x with PBS. Enteroids were permeabilized and stained in phosphate-buffered saline with 3% bovine serum albumin, 1% Triton X-100, and 1% saponin. Images were collected using a 20x or 40x oil immersion objective on an LSM 700 confocal microscope (Carl Zeiss) with Zen 2009 software (Carl Zeiss). Images were 3D-reconstructed using Volocity Image Analysis software (Improvision). Samples were stained with 40,6-Diamidino-2-phenylindole (DAPI) and either AlexaFluor 488 phalloidin, AlexaFluor 594 phalloidin, or AlexaFluor 660 phalloidin (Invitrogen) for visualization of the nuclei and actin. All antibodies were diluted 1:100, except antibodies against MUC2 and villin which were diluted 1:200.

Antibody treatments

BME-embedded enteroids were treated with no antibody, anti-β1 integrin-neutralizing antibody clone AIIB2 (Developmental Studies Hybridoma Bank), or a control antibody against intracellular antigen LAMP-1 clone H4A3 (Developmental Studies Hybridoma Bank). Antibodies were diluted to 2.2 μg/mL in growth media and added to enteroids for 1 day. Treated enteroids were analyzed by confocal microscopy to determine the frequency of basal-out, apical-out, and mixed polarity enteroids.

qRT-PCR

BME-embedded or suspended enteroids were collected and pelleted after incubation in growth media, or after 3-5 days in differentiation media. RNA was isolated from the enteroids using the RNeasy Plus Micro Kit (QIAGEN). cDNA was synthesized from RNA using the SuperScript III First-Strand Synthesis System (Invitrogen). All samples were analyzed on an Applied Biosystems 7300 real-time PCR system with the FastStart Universal SYBR Green Kit (Roche). Reaction conditions were 50°C for 2 min; 95°C for 10 min; 40 cycles of 95°C for 15 s and 60°C for 1 min; followed by a dissociation stage of 95°C for 15 s, 60°C for 1 min, 95°C for 15 s, and 60°C for 15 s. Primers for the following genes GAPDH, CHGA, LYZ, MUC2, SI were from VanDussen et al. (2015) and primers for LGR5 were from Bartfeld et al. (2015).

Epithelial barrier integrity

Apical-out enteroids (cultured in suspension with growth media for 1-3 days) were pelleted and re-suspended in a solution of 4 kDa FITC-Dextran (2 mg/mL diluted in growth media). As a control for disrupted barrier integrity, apical-out enteroids were treated with 2 mM EDTA in HBSS (with no calcium and no magnesium) on ice for 15 min, then re-suspended in the FITC-Dextran solution. Enteroids in the FITC-dextran solution were mounted onto a slide and immediately imaged live by

DIC and fluorescence microscopy using a 20x objective on an LSM 700 confocal microscope (Carl Zeiss) with Zen 2009 software (Carl Zeiss).

To interrogate epithelial barrier integrity after bacterial infection, enteroids were mock infected (media only control) or infected for 1 h with *S.* Typhimurium SL1344-mCherry. Prior to infection, bacteria were subcultured from an overnight LB culture 1:20 into LB with 300 mM NaCl and grown standing at 37°C with 5% CO_2 for 2-3 h. Enteroids were pelleted by gravity in a microcentrifuge tube, then resuspended in the FITC-dextran solution, mounted onto a slide and immediately imaged live by confocal microscopy as described above. The percentage of enteroids with intact barrier integrity was determined by dividing the number of enteroids that excluded FITC-dextrans by the total number of enteroids. 5 different experiments were analyzed using enteroids derived from 2 different donors.

Fatty acid absorption

Apical-out or basal-out enteroids were tested for fatty acid absorption. For basal-out enteroids, BME-embedded enteroids were solubilized in 5 mM EDTA in PBS on a rotating platform at 4°C for 1 h. For apical-out enteroids, enteroids were incubated in suspension culture in growth media for 3 days. Enteroids were washed with DMEM with no phenol red, then re-suspended in a solution of 5 μM fluorescent fatty acid analog C1-BODIPY-C12 with 5 μM fatty-acid-free BSA. Enteroids in the fatty acid analog solution were plated in low attachment 24-well plates and incubated for 30 minutes. Enteroids were fixed in 2% paraformaldehyde in 100 mM phosphate buffer (pH 7.4) and imaged using confocal microscopy. Enteroids were stained for nuclei and actin, and a single confocal z-scan was taken for each enteroid. The intracellular fluorescent signal from absorbed C1-BODIPY-C12 was quantified using FIJI (ImageJ). 10 enteroids were analyzed per well using the same acquisition settings. 4 different experiments were analyzed using enteroids derived from 3 different donors.

Time lapse microscopy of immobilized apical-out enteroids

BME-embedded enteroids were dislodged with a sterile spatula and solubilized in 5 mM EDTA in PBS for 1 h at 4°C on a rotating platform. 10 μL BME was added into the wells of a Nunc Lab-Tek II 2-well chambered coverglass wells, and spread with a sterile spatula to form a thin layer. The BME layer was polymerized at 37°C for 10 minutes. Enteroids were centrifuged at 200 x g for 3 min at 4°C and the supernatant was removed. The enteroid pellet was re-suspended in 30 μl media and spotted onto the BME layer. Enteroids were allowed to attach for 15 minutes in the incubator, then media was added to fill the chamber. The enteroids were monitored using a Zeiss Axiovert 200M microscope using a 10x differential interference contrast (DIC) microscopy objective. Samples were kept at 37°C with 5% CO_2 during imaging. Time-lapse images were collected by a Hamamatsu ORCA-100 C4742-5 digital camera and stored digitally every 30 minutes using OpenLab 5.5.2 software (Improvision). After recording, the images were collated into a digital movie sequence using FIJI (ImageJ). Samples were fixed and stained for immunofluorescence confocal microscopy.

Enteroid infections

For basal-out enteroids, BME-embedded enteroids were solubilized in 5 mM EDTA in PBS on a rotating platform at 4°C for 1 hour, then washed in DMEM immediately prior to infections. For apical-out enteroids, enteroids were incubated in suspension culture for 3 days prior to infection. Enteroids were centrifuged at 300 x g for 3 minutes, supernatants were removed, then enteroids were resuspended in media containing bacteria. Bacteria were grown as described above, and pelleted at 6000 x g for 3 minutes. *S.* Typhimurium-mCherry was re-suspended in Advanced DMEM/F-12 to OD_{600} 0.01. Bacteria were allowed to invade for 1 hour, then the enteroids were either fixed or isolated and transferred to media containing gentamicin (100 μg/mL) for 1 hour to kill residual extracellular bacteria. Media was then replaced with low gentamicin (10 μg/mL) media for the remainder of the infection. For *L. monocytogenes* infections, *L. monocytogenes*-GFP was re-suspended in DMEM to OD_{600} 0.1. Enteroids were incubated with *L. monocytogenes* for 15 minutes, then washed twice with DMEM and fixed or re-suspended in DMEM. Attached *L. monocytogenes* were allowed to invade until 1 hour post-infection, then extracellular bacteria were killed with gentamicin (50 μg/mL) for 20 minutes. Media was changed to low gentamicin (10 μg/mL) media for the remainder of the experiment. Infected enteroids were fixed, stained, and imaged using confocal microscopy. Invaded/invading *S.* Typhimurium or attached *L. monocytogenes* were quantified by manual counting of bacteria in confocal Z stacks of 10 enteroids in 3 experiments, using enteroid lines derived from 3 different donors.

QUANTIFICATION AND STATISTICAL ANALYSIS

Data are expressed as mean values ± standard deviations, which were calculated from at least 3 experiments. In graphs, each point represents an individual enteroid. Statistical analyses were performed using GraphPad Prism 7 software, with significance set at $p < 0.05$. Statistical tests, *n,* and p-values are indicated in figure legends.

Cell Reports, Volume *26*

Supplemental Information

Controlling Epithelial Polarity:

A Human Enteroid

Model for Host-Pathogen Interactions

Julia Y. Co, Mar Margalef-Català, Xingnan Li, Amanda T. Mah, Calvin J. Kuo, Denise M. Monack, and Manuel R. Amieva

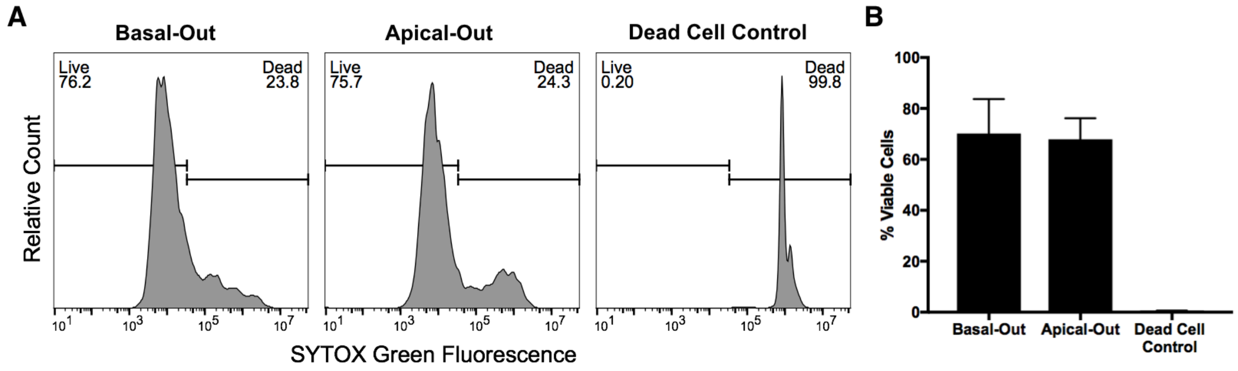

Figure S1. Flow cytometry analysis shows comparable cell viability for basal-out and apical-out enteroids, related to Figure 1. (**A**) Representative histograms of basal-out enteroid cells, apical-out enteroid cells, or ethanol-killed enteroid cells labeled with SYTOX Green dead cell stain. (**B**) Quantification of basal-out enteroid cells, apical-out enteroid cells, or ethanol-killed enteroid cells labeled with SYTOX Green dead cell stain. Data represented are mean ± SD; n = 3 experiments.

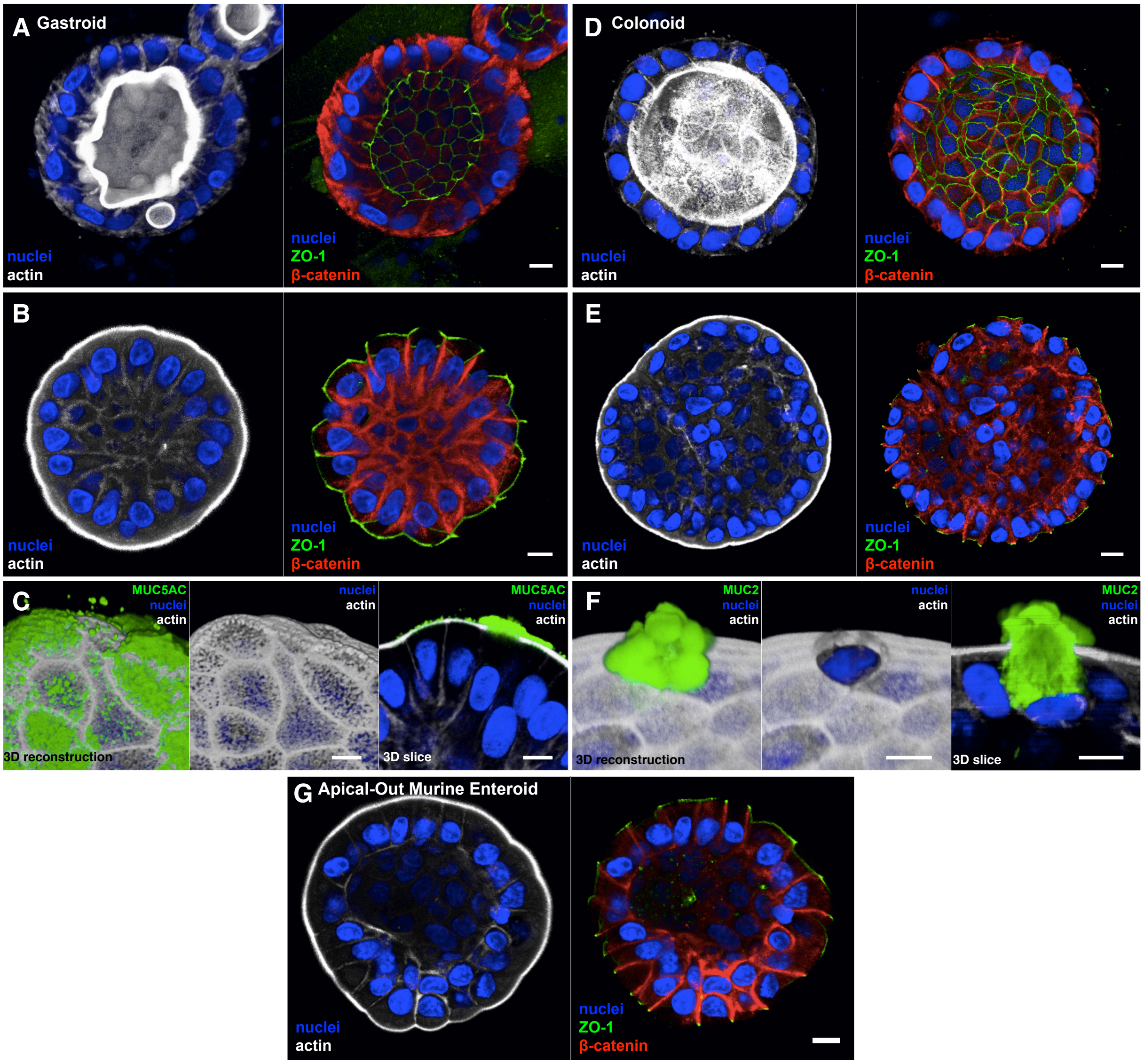

Figure S2. Polarity reversal occurs in human gastroids, human colonoids, and murine enteroids, related to Figure 1. Confocal microscopy shows that (**A**) BME-embedded gastroids exhibit basal-out polarity and (**B**) suspended gastroids have apical-out polarity. Nuclei in blue, actin in white, ZO-1 in green, and β-catenin in red are shown. (**C**) Apical-out gastroid cells produce the gastric mucin MUC5AC (green). (**D**) BME-embedded colonoids have basal-out polarity and (**E**) suspended colonoids have apical-out polarity. Nuclei in blue, actin in white, ZO-1 in green, and β-catenin in red are shown. (**F**) Apical-out colonoids can differentiate to goblet cells which secrete the intestinal mucin MUC2 (green). (**G**) Murine enteroids in suspension culture have apical-out polarity. Nuclei in blue, actin in white, ZO-1 in green, and β-catenin in red are shown. All scale bars are 10 µm.

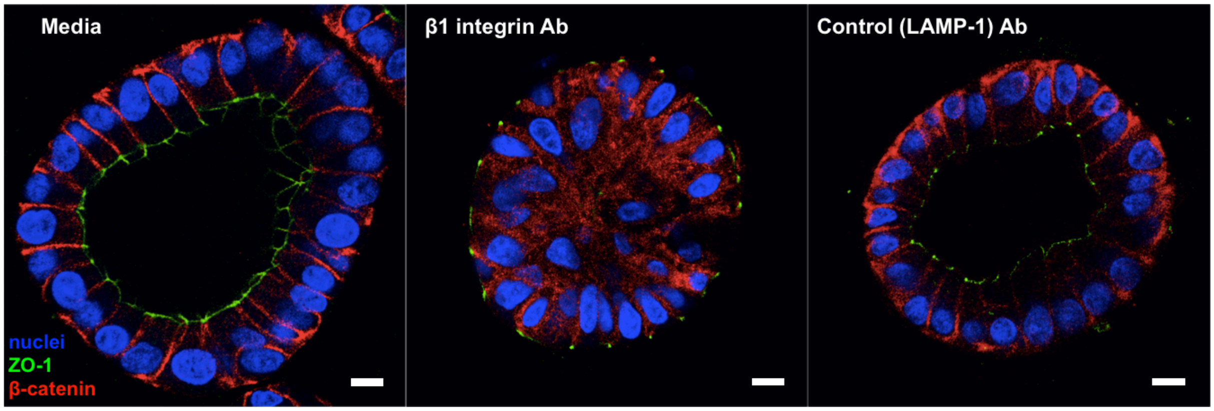

Figure S3. β1-integrin regulates enteroid polarity, related to Figure 2. Representative confocal images of BME-embedded enteroids incubated in media alone, with β1-integrin function-blocking antibody, or a control antibody for 1 day. Nuclei in blue, actin in white, ZO-1 in green, and β-catenin in red are shown. Scale bars are 10 µm.

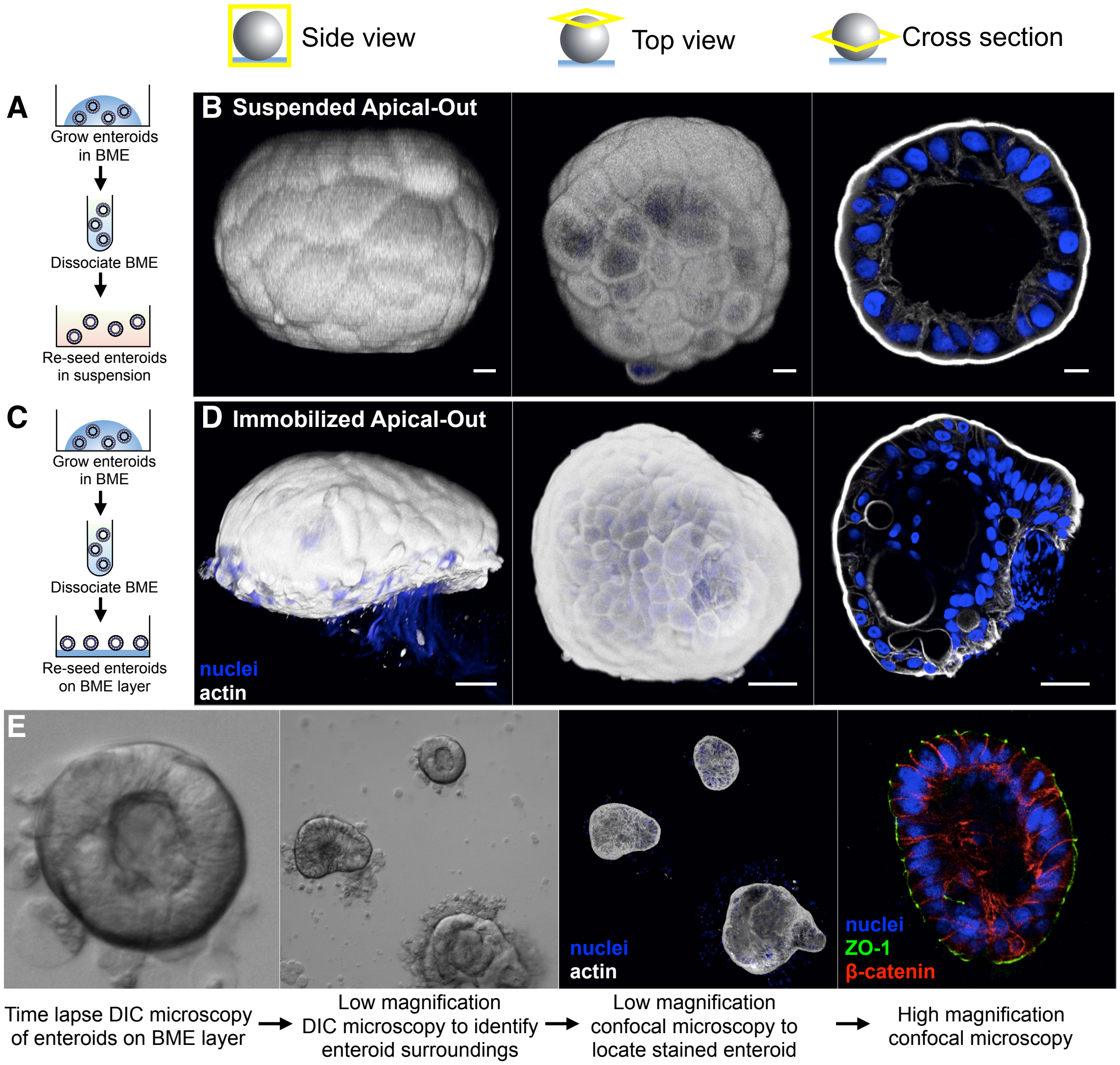

Figure S4. Immobilized apical-out enteroids for retrospective fluorescence time-lapse microscopy, related to Figure 1, 2. Comparison of (**A, B**) suspended and (**C**, **D**) immobilized apical-out enteroids. (**A**) Schematic for generating suspended apical-out enteroids. (**B**) Confocal microscopy of suspended enteroids, which have complete apical-out polarity. Scale bars are 10 µm. (**C**) Schematic to produce immobilized apical-out enteroids. (**D**) Confocal microscopy of immobilized apical-out enteroids, which have apical-out polarity except at the regions where the enteroid is in contact with the BME. Scale bars are 50 µm. (**E**) Workflow for retrospective fluorescence time-lapse microscopy. An enteroid is first imaged using time-lapse DIC microscopy, located within its surroundings, then fixed, stained and located using confocal microscopy, and finally imaged by confocal microscopy at higher magnification. For confocal images, nuclei in blue, actin in white, ZO-1 in green, and β-catenin in red are shown.

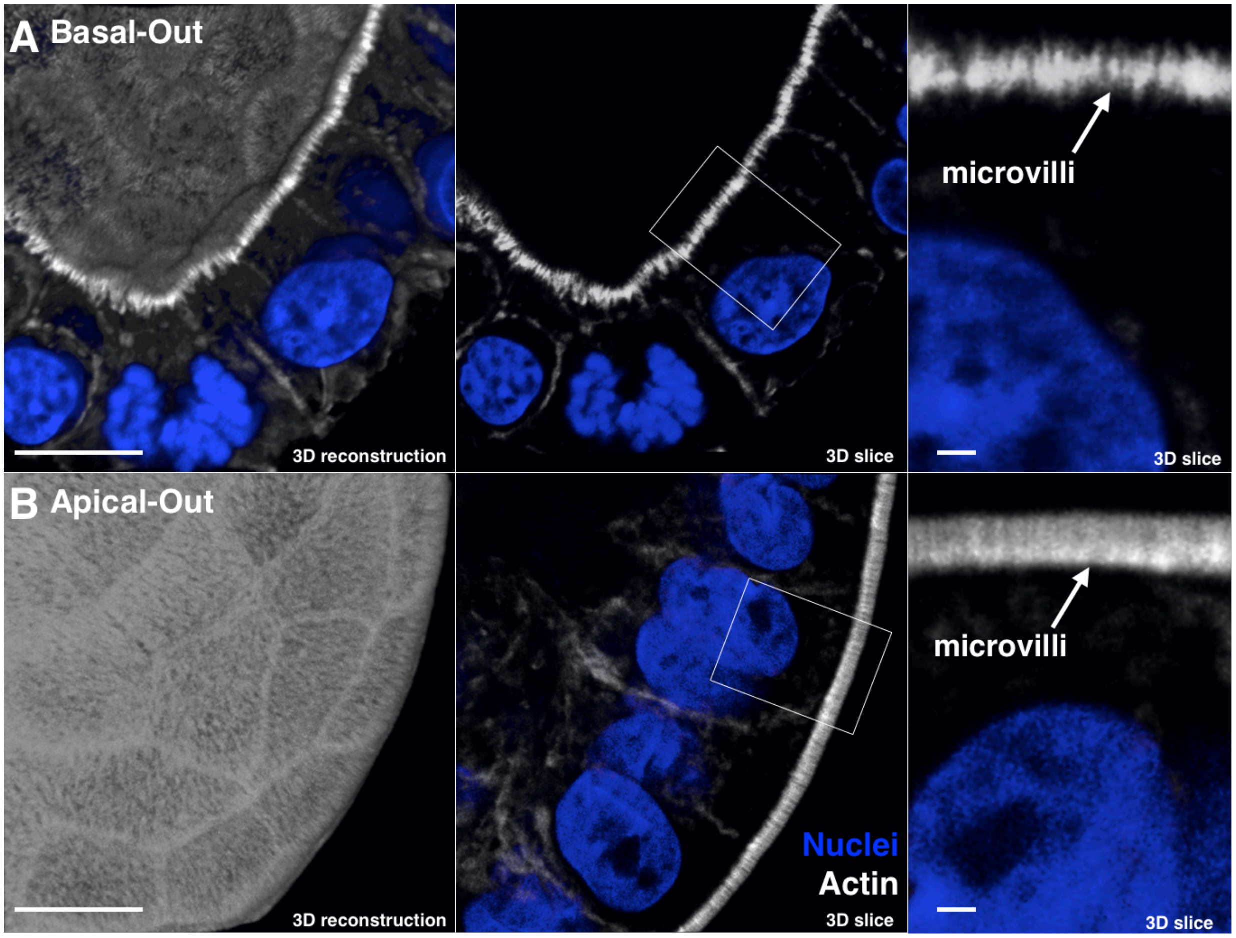

Figure S5. Apical microvillar structures of basal-out enteroids and apical-out enteroids, related to Figure 4. **(A)** Confocal microscopy of a BME-embedded basal-out enteroid shows the actin-rich apical microvilli are on the lumenal surface of the enteroid. **(B)** For apical-out enteroids, the actin-rich microvilli are on the outer enteroid surface. Left panels are 3D reconstructions and center panels are 3D slices through the cells (scale bars are 10 µm). Right panels are magnified insets of the middle panels (scale bars are 1 µm). Nuclei in blue and actin in white are shown.

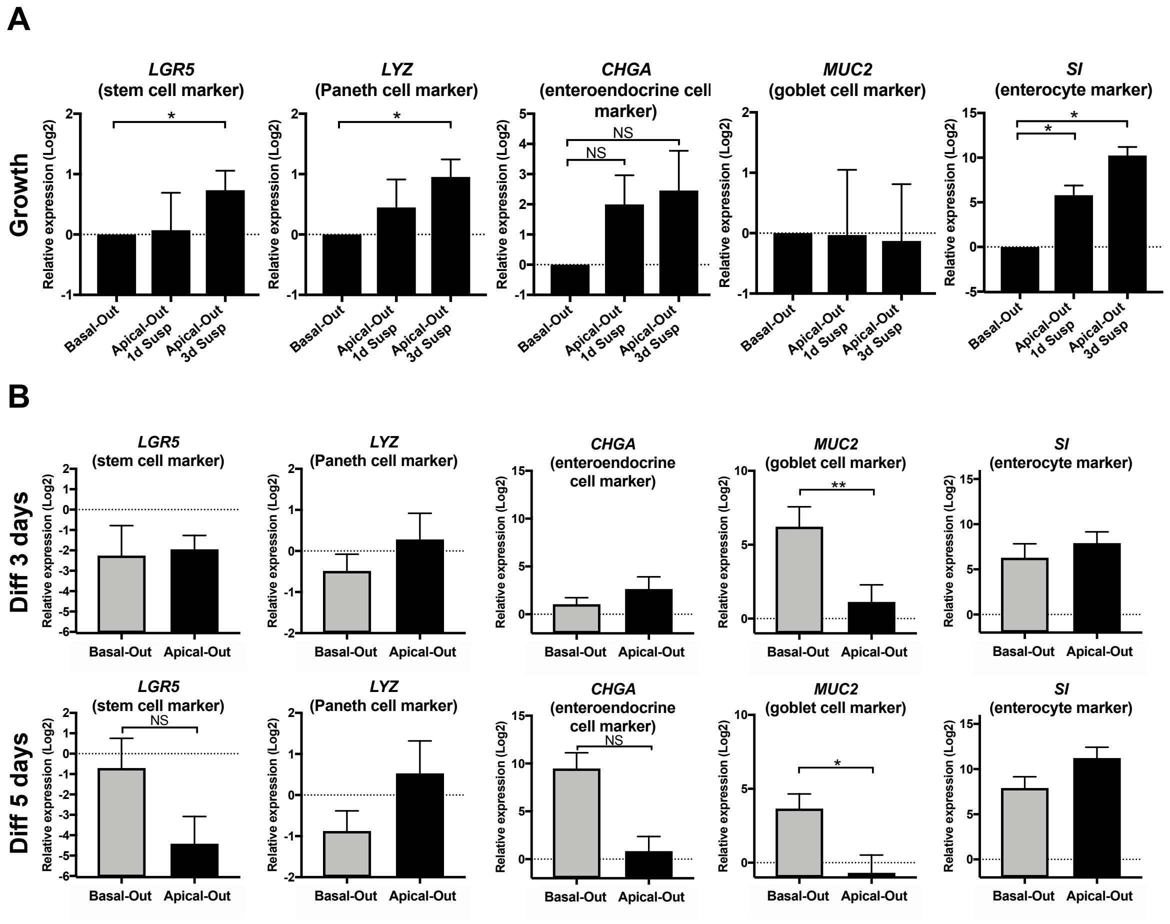

Figure S6. qRT-PCR gene expression analysis of enteroids, related to Figure 4. Expression of stem cell marker *LGR5* (which encodes leucine-rich repeat-containing G-protein coupled receptor 5), Paneth cell marker *LYZ* (which encodes lysozyme), enteroendocrine marker *CHGA* (which encodes chromagranin A), goblet cell marker *MUC2* (which encodes mucin 2), and enterocyte marker *SI* (which encodes sucrase isomaltase) was evaluated for enteroids cultivated in the following conditions: (**A**) In growth media, BME-embedded basal-out enteroids or suspended apical-out enteroids cultured for 1 or 3 days. Values are reported as fold-change relative to expression in basal-out enteroids cultured in growth media (dotted lines). Data represented are mean ± SD; n ≥ 3 experiments; *p < 0.05 using the Kruskal-Wallis test with Dunn's multiple comparison test. (**B**) In differentiation media, BME-embedded basal-out enteroids or suspended apical-out enteroids cultured for 3 days or 5 days. Values are reported as fold-change relative to expression in basal-out enteroids cultured in growth media (dotted lines). Data represented are mean ± SD; n ≥ 4 experiments; *p < 0.05, **p < 0.05 using the Mann-Whitney U test.

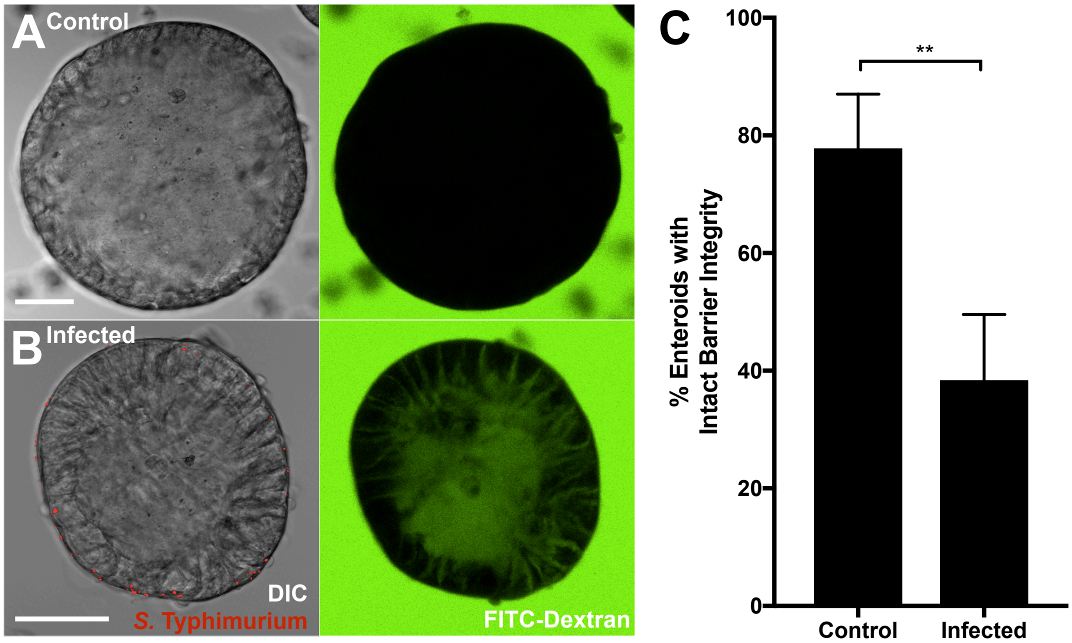

Figure S7. *S.* Typhimurium infection disrupts epithelial barrier integrity in apical-out differentiated enteroids, related to Figure 6. The dextran diffusion assay was used to evaluate uninfected (control) apical-out enteroids, or apical-out enteroids infected with *S.* Typhimurium for 1 hour. (**A**) An uninfected (control) apical-out enteroid that has intact barrier integrity. (**B**) An infected apical-out enteroid with disrupted barrier integrity. Scale bars are 50 µm. (**C**) Quantification of uninfected (control) or infected enteroids that exclude FITC-dextrans and thus have intact barrier integrity. Data represented are mean ± SD; n = 5 experiments; $p < 0.01$ using the Mann-Whitney U test.
